# Improved electrochemical conversion of CO_2_ to multicarbon products by using molecular doping

**DOI:** 10.1038/s41467-021-27456-5

**Published:** 2021-12-10

**Authors:** Huali Wu, Ji Li, Kun Qi, Yang Zhang, Eddy Petit, Wensen Wang, Valérie Flaud, Nicolas Onofrio, Bertrand Rebiere, Lingqi Huang, Chrystelle Salameh, Luc Lajaunie, Philippe Miele, Damien Voiry

**Affiliations:** 1 Institut Européen des Membranes, IEM, UMR 5635, Université Montpellier, ENSCM, CNRS, Montpellier, 34000 France; 2 College of Bioresources and Materials Engineering, Shaanxi University of Science & Technology, 710021 Xi’an, People’s Republic of China; 3 Institut Charles Gerhardt, ICGM, UMR 5253, University of Montpellier, ENSCM, CNRS, 34095 Montpellier Cedex 5, France; 4 School of Science and Engineering, The Chinese University of Hong Kong, 518172 Shenzhen, Guangdong People’s Republic of China; 5 Departamento de Ciencia de los Materiales e Ingeniería Metalúrgica y Química Inorgánica, Facultad de Ciencias, Universidad de Cádiz, Campus Río San Pedro S/N, Puerto Real, 11510 Cádiz, Spain; 6 Instituto Universitario de Investigación de Microscopía Electrónica y Materiales (IMEYMAT), Facultad de Ciencias, Universidad de Cádiz, Campus Río San Pedro S/N, Puerto Real, 11510 Cádiz, Spain; 7 Institut Universitaire de France (IUF), 1 rue Descartes, 75231 Paris Cedex 05, France

**Keywords:** Electrocatalysis, Electrocatalysis, Electrocatalysis

## Abstract

The conversion of CO_2_ into desirable multicarbon products via the electrochemical reduction reaction holds promise to achieve a circular carbon economy. Here, we report a strategy in which we modify the surface of bimetallic silver-copper catalyst with aromatic heterocycles such as thiadiazole and triazole derivatives to increase the conversion of CO_2_ into hydrocarbon molecules. By combining operando Raman and X-ray absorption spectroscopy with electrocatalytic measurements and analysis of the reaction products, we identified that the electron withdrawing nature of functional groups orients the reaction pathway towards the production of C_2+_ species (ethanol and ethylene) and enhances the reaction rate on the surface of the catalyst by adjusting the electronic state of surface copper atoms. As a result, we achieve a high Faradaic efficiency for the C_2+_ formation of ≈80% and full-cell energy efficiency of 20.3% with a specific current density of 261.4 mA cm^−2^ for C_2+_ products.

## Abstract (web summary)

Strategies to systematically tune CO_2_ electroreduction to multicarbon products are of high interests. Here the authors report electron withdrawing functional group alters the reaction pathway towards C_2+_ products by adjusting the oxidation state of surface copper.

## Introduction

The rapid increase in the atmospheric carbon dioxide (CO_2_) levels has motivated the development of carbon capture, utilization, and storage (CCUS) technologies. In this context, the electrochemical reduction of CO_2_ to hydrocarbons using renewable energy is regarded as an effective way to close the carbon cycle via the conversion of CO_2_ into chemical precursors or fuels^1,2^. The electrochemical CO_2_ reduction reaction (CO_2_RR) toward single carbon products has achieved enormous progress^3^, especially for the production of C_1_ molecules such as carbon monoxide (CO) or methane (CH_4_)^4–7^. Copper (Cu) is one of the few transition metals that can efficiently catalyze the electrolysis of CO_2_ to multicarbon products such as ethylene, ethanol, acetate, propanol^8^. Because multicarbon products possess higher market values and are more energy concentrated^1^, intensive efforts have been devoted to improve the reaction selectivity towards the production of C_2_ and C_2+_ molecules. Examples of strategies for optimizing the Faradaic efficiency towards the production of C_2+_ species include alloying^9–12^, surface doping^13,14^, ligand modification^15,16^, and interface engineering^17–20^.

Designing Cu-based catalysts by adapting some of the concepts of molecular catalysts in order to finely tailor the behavior of the active sites of metallic surfaces is currently regarded as the long-standing interest for the controlled design of novel electrocatalytic materials. Increasing the oxidation state of copper has been suggested to improve the CO_2_RR performance and notably the formation of C_2+_ species^14,21,22^. Various strategies are being explored to prepare Cu^δ+^ by using controlled oxidation via plasma treatments or doping with boron and halides^14,23–25^. Alternatively, molecular engineering of either the electrolyte or the catalyst surface has recently been proposed for orienting the selectivity of the reaction by stabilizing intermediates, inhibiting proton diffusion, or acting as redox mediators during the electrochemical CO_2_ reduction reaction (CO_2_RR)^26–30^. Organic species such as N-aryl pyridinium salts^31,32^, imidazole^33–35^, thiol^36,37^, and cysteamine^38^ have been reported as an effective lever to tune the reaction selectivity toward the formation of specific products by stabilizing key reaction intermediates. Functionalization of alkyl chains can also lead to better CO_2_RR performance by suppressing the competitive hydrogen evolution reaction (HER) via the creation of hydrophobic regions on the surface of the catalyst^37,39,40^.

Here we present an effective strategy to control the surface oxidation state of bimetallic Ag–Cu electrodes by using functionalization for tuning the oxidation state of Cu^δ+^. By combining Auger and X-ray absorption spectroscopies (XAS), we identified that the grafting of aromatic heterocyclic functional groups can efficiently dope the surface of Cu by withdrawing electrons from the metal surface leading to the formation of Cu^δ+^ species. Compared to pristine non-functionalized and alkyl-functionalized electrodes, the modified electrodes display a clear improvement of the reaction rates and Faradaic efficiency towards the production of C_2+_ products. Operando Raman and X-ray absorption spectroscopy (XAS) suggest that the presence of Cu^δ+^ with 0 < δ < 1—due to the *p*-doping of the Cu surface—favors the formation of adsorbed CO with the atop conformation which is a known key intermediate species involved in the C–C coupling step associated with the formation of multicarbon products. When assembled in a membrane electrode-assembly electrolyzer, the catalyst delivers a Faradaic efficiency (FE) for C_2+_ products of 80 ± 1% and a total C_2+_ energy efficiency (EE) of 20.3% for the full cell.

## Results

### Catalyst design and characterization

**Fig. 1:**
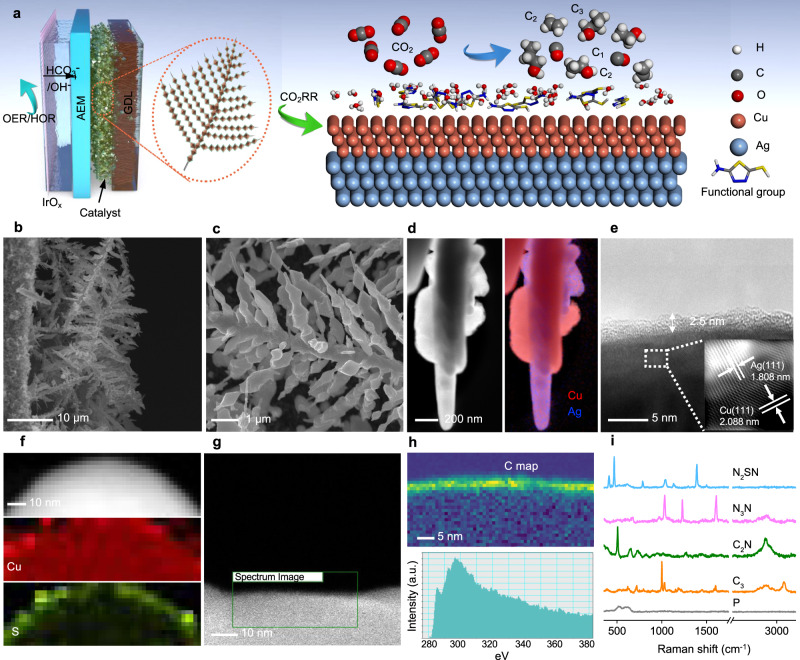
Structural and elemental composition of the functionalized Ag–Cu catalysts. **a** Schematic representation of the functionalized Ag–Cu electrodes in a membrane electrode assembly. **b**, **c** Cross-section (**b**) and top-view (**c**) scanning electron microscope (SEM) images of the functionalized hierarchical Ag–Cu catalyst on a gas diffusion electrode (GDE). **d** High-angle annular dark-field scanning transmission electron microscopy (HAADF-STEM) image (left) and corresponding Cu (red) and Ag (blue) EDS elemental maps of N_2_SN-functionalized Ag–Cu (right). **e** High-resolution transmission electron microscope (HR-TEM) micrograph of the N_2_SN-functionalized electrode (**e**). **f** HAADF-STEM image and the corresponding Cu and S EDS elemental maps taken from a section of Cu surface on the N_2_SN-functionalized Ag–Cu electrode. **g** HAADF-STEM image of the Cu surface of N_2_SN-functionalized Ag–Cu. **h** (top), Electron energy-loss spectroscopy (EELS) elemental mapping of C taken from the area marked by the box in **g**. **h** (bottom), EELS spectrum of the C-K edge with fine structures characteristics of carbon linked to heteroatoms from N_2_SN layer on the Cu surface. **i** Raman spectra of pristine (non-functionalized) Ag–Cu (gray), C_3_-functionalized Ag–Cu (orange), C_2_N-functionalized Ag–Cu (green), N_3_N-functionalized Ag–Cu (purple) and N_2_SN-functionalized Ag–Cu (blue).

We fabricated the functionalized bimetallic catalyst by using a two-step strategy based on the controlled electrodeposition of Ag and Cu followed by the modification of the catalyst surface via functionalization (Fig. 1a). The Ag–Cu electrodes were prepared by firstly depositing Ag on gas diffusion electrodes (GDE) using pulsed electrodeposition. The silver structure grows in the form of a dendritic fish-bone structure with sharp Ag nanoneedles (Supplementary Fig. 1). The Ag layer was then used as a scaffold for the deposition of copper. The final structure of the catalyst on the GDE electrodes is found to be porous where Cu is preferentially deposited on Ag (Fig. 1b, c and Supplementary Fig. 2). The catalytic performance of pure Cu and Ag–Cu electrodes were systematically investigated (Supplementary Figs. 2 and 3), and our results indicated that appropriate loading of Ag contributes to the enhancement of the formation of CO, which may further facilitate C_2+_ production on copper. And the optimum composition is 15%_at._ Ag in Ag–Cu (labeled as 15%_at._ Ag–Cu). To control the oxidation state of Cu, we sought to functionalize the catalyst with thiol molecules via dip coating. We selected thiadiazole (N_2_SN) and triazole (N_3_N) derivatives as electron-deficient functional molecules to react with the surface of the catalyst^41–44^ (Supplementary Fig. 4). For comparison, the bimetallic electrodes were also modified with 1-propanethiol (C_3_) and cysteamine (C_2_N) as model short alkyl and alkyl amine functional groups (Supplementary Fig. 4 and Supplementary Figs. 5c, d). The modification of the electrode is clearly visible from the change of the water contact angle that varies between 86° and 129° depending on the nature of the functional groups compared to 84° for the pristine catalyst (Supplementary Fig. 6). To verify the presence of the functional groups, we performed energy-dispersive X-ray spectroscopy (EDS) analyses in a SEM. The corresponding elemental maps at low magnification show the uniform distribution of S, N, and C on Ag–Cu electrode. A thin amorphous layer is observed under high-resolution TEM at the surface of the catalyst with a thickness of ≈2.5 nm which also corresponds to an increase of the S signal in the corresponding EDS elemental map (Figs. 1d–f and Supplementary Fig. 7). The existence of an organic layer on the Ag–Cu electrodes is further confirmed by the high-angle annular dark-field scanning transmission electron microscopy (HAADF-STEM) and the electron energy-loss spectroscopy (EELS) mapping of the carbon element. Remarkably, the EELS spectrum of the C-K edge displays fine structural characteristics of carbon linked to heteroatoms at ≈292 eV (Figs. 1g, h and Supplementary Fig. 8). Raman and Fourier transformed infrared (FTIR) spectroscopies were also used to further confirm the successful attachment of the functional groups on the surface of the catalyst (Fig. 1i and Supplementary Fig. 9). The Raman signatures of the different grated molecules were detected on the surface of the Ag–Cu electrodes, while strong FTIR bands at 1303, 1584, and 1622 cm^−1^ are only presented on N_2_SN-, N_3_N- and C_2_N-functionalized Ag–Cu electrodes and attributed to the C–C or C–N stretching, the NH_2_ scissor and the C–N stretching modes respectively^45–47^ (Supplementary Fig. 9). The successful functionalization with thiadiazole and triazole is further confirmed from the deconvolution of the X-ray photoelectron spectra from the S2*p* and N1*s* regions respectively (Supplementary Figs. 10b, c). The peak of S2*p* was deconvoluted into three doublets at 162.75, 164.23, and 168.31 eV for the S2*p*_3/2_, corresponding to S–H and S–C bonds on both thiadiazole and triazole, respectively^48^. Analogously, the N1*s* spectrum (Supplementary Fig. 10c) can be divided into three components at 398.24, 399.63, and 400.70 eV, which reflects the existence of N–N, C–N, and N–H bonds on the surface of functionalized electrodes. The presence of crystalline Ag and Cu on the gas diffusion electrode was further observed from the X-ray diffraction patterns, whereas the presence of distinct peaks from the Ag and Cu facets agrees with the absence of alloy structure of the bimetallic catalyst (Supplementary Fig. 11). To clarify the orientation of the aromatic heterocycles on the catalyst surface, we carried out density functional theory (DFT) calculations to estimate the total energy and the binding energy of thiadiazole on Cu using a model with 5 Cu (111) slabs (Supplementary Figs. 12 and 13). Among the different configurations tested, the adsorption of thiadiazole is more stable when the N_2_–N_3_ nitrogen atoms of the diazole sit on Cu (111) and the binding energy is estimated to be −1.08 eV—at least 0.37 eV lower than for the other configurations (Supplementary Table 1).

### CO_2_ electroreduction performance in H cell

**Fig. 2:**
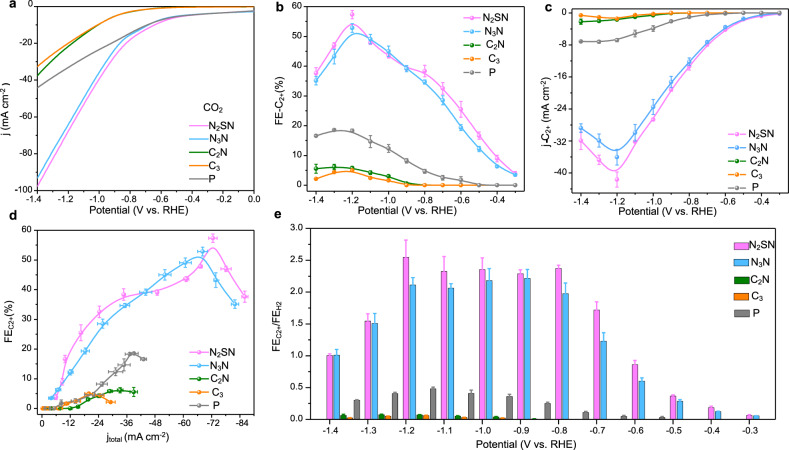
CO_2_RR performance of the functionalized Ag–Cu electrodes in a H-cell. **a** Linear scan voltammetry (LSV) curves measured for different samples: N_2_SN, N_3_N, C_2_N, C_3_ functionalized Ag–Cu compared to pristine (P) Ag–Cu in CO_2_-saturated 0.5 M KHCO_3_ at electrochemical potential (V) from 0 to −1.4 V vs. RHE. Scan rate, 20 mV s^−1^. **b** Faradaic efficiency (FE) values for C_2+_ products on different samples at various potentials ranging from −0.3 to −1.4 V vs. RHE and measured in 0.5 M KHCO_3_. **c** *j*–*V* plots of the partial current densities for the C_2+_ products (ethylene and ethanol). **d** Relationships between the FE for C_2+_ and the total current density for all the catalysts. (**e**), Selectivity for C_2+_ products over hydrogen based on the ratio in FEs of C_2+_ and hydrogen. The error bars in **b**–**e** correspond to the standard deviation of three independent measurements.

The functionalized electrodes were electrochemically tested in a H-cell reactor using Argon and CO_2_-saturated 0.5 M KHCO_3_ electrolyte solutions. Figure 2a shows that thiadiazole (N_2_SN) and triazole (N_3_N) functionalized electrodes exhibit the highest current density and lowest onset potential in CO_2_-saturated solution. We then evaluated the Faradaic efficiency (FE) by using nuclear magnetic resonance (NMR) and gas chromatography (GC) (See details in the Methods section). H_2_, CO, formate, CH_4_, and C_2+_ products were formed on the bimetallic electrode (Supplementary Fig. 14). Remarkably, the Faradaic efficiency for C_1_ and H_2_—obtained via the CO_2_RR and HER—decreased after functionalization with thiazole and thiadiazole, while the FE for C_2+_ products sharply increases (Fig. 2b). Ethylene and ethanol are the major C_2+_ products detected, together with a trace amount of acetate and n-propanol (Supplementary Figs. 14 and 15). The FE for C_2+_ on N_2_SN- and N_3_N-functionalized electrodes are estimated to 57.3% and 51.0% at −1.2 V versus the reversible hydrogen electrode (vs. RHE) compared to only 18% for the pristine catalyst corresponding to enhancements of 3.1 and 2.8 folds respectively (Fig. 2b). The selectivity towards the formation of C_2+_ products for both thiazole and thiadiazole functional groups increases continuously with increasing voltage from −0.3 to −1.2 V vs. RHE and starts decreasing after −1.3 V, whereas the values of FE for C_1_ products and H_2_ exhibit a volcano-shaped dependence with the applied potentials (Supplementary Figs. 16a, b). This leads to an obvious enhancement of the specific current density for C_2+_ products (${{{{{{\rm{j}}}}}}}_{{{{{{{\rm{C}}}}}}}_{2}+}$) up to 5 folds at −1.2 V vs. RHE (Fig. 2c). Conversely, the functionalization of the Ag–Cu electrodes with short alkyl or aminoalkyl chains does not suppress the HER pathway nor improve the CO_2_RR activity (Fig. 2d). C_2_N- and C_3_- modified catalysts clearly display lower activities towards the CO_2_RR, notably with minimal production of C_2+_ species and a relatively large FE for the evolution of H_2_. Our results, therefore, highlight the importance of the nature of the functional groups on the CO_2_RR performance. To better evaluate the selectivity of C_2+_ products on thiadiazole- and triazole-functionalized Ag–Cu electrodes, we calculated the ratio in FE for C_2+_ products and hydrogen (${{{{{\rm{F}}}}}}{{{{{{\rm{E}}}}}}}_{{{{{{{\rm{C}}}}}}}_{2+}}/{{{{{\rm{F}}}}}}{{{{{{\rm{E}}}}}}}_{{{{{{{\rm{H}}}}}}}_{2}}$) (Fig. 2e). Compared with pristine and alkyl-functionalized electrodes, both N_2_SN and N_3_N functional groups present the largest ${{{{{\rm{F}}}}}}{{{{{{\rm{E}}}}}}}_{{{{{{{\rm{C}}}}}}}_{2+}}/{{{{{\rm{F}}}}}}{{{{{{\rm{E}}}}}}}_{{{{{{{\rm{H}}}}}}}_{2}}$ ratios—illustrating that the functionalization with aromatic heterocycles efficiently directs the reaction pathway towards the formation of C_2+_ products while suppressing the HER. To get a more accurate estimation of the intrinsic CO_2_RR performance of the functionalized Ag–Cu electrodes, we estimated the electrochemically active surface area of Cu (Cu ECSA) and Ag (Ag ECSA) for the 15 at.% Ag–Cu and the N_2_SN-15 at.% Ag–Cu catalysts using Pb underpotential deposition (Pb UPD) (Supplementary Figs. 17, 18 and Supplementary Table 2). The partial current densities for C_2+_ products measured in H-cell were normalized by the ECSA values for Cu. Remarkably, we found that the ECSA-normalized partial current density on N_2_SN functionalized Ag–Cu is 5.3 mA cm^−2^, which is around five times larger than that for the pristine 15 at.% Ag–Cu catalyst (Supplementary Fig. 18). Electrochemical impedance spectroscopy (EIS) measurements were performed to explore the charge transfer processes on the surface of the different electrodes during the electrolysis of CO_2_. The charge transfer resistance of the N_2_SN- and N_3_N- functionalized electrodes is not substantially perturbed compared to that of the pristine bimetallic catalyst (Supplementary Fig. 19). On the contrary, the resistance is significantly larger in the case of electrodes functionalized with 1-propanthiol and cysteamine indicating that the charge transfer is strongly affected; likely due to the strong hydrophobicity of the surface of the alkyl-functionalized catalyst.

To gauge the stability of the functionalization, we operated the electrodes at a potential of −1.2 V vs. RHE for more than 20 h in the H-cell reactor, while recording the current density and continuously analyzing the products of the reaction (Supplementary Fig. 20). The N_2_SN- and N_3_N-functionalized electrodes demonstrated stable performance with a retention of the current density of 94% and 91% respectively—sharply improved compared to that of pristine Ag–Cu at 78%. The FE for C_2+_ of N_2_SN and N_3_N functionalized Ag–Cu electrodes remains as high as 54% and 46.5% after 20 h, which demonstrates that the selectivity for the reaction pathway on the surface of the electrode is not modified during electrolysis. To further confirm the apparent stability of the functionalized electrode, we performed XPS spectroscopy to evaluate the N:Cu ratio after 30 min, 1 h, 24 h, and 100 h. The ratio is found to be virtually constant suggesting a robust grafting of the functional groups on the catalyst surface (Supplementary Figs. 21, 22 and Supplementary Table 3).

### Ex situ and in situ mechanistic investigations

**Fig. 3:**
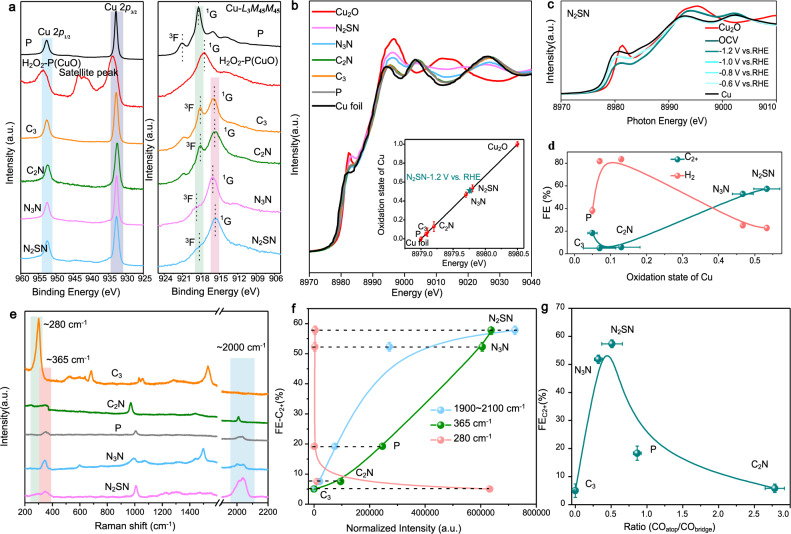
Physical characterizations of the functionalized electrodes using XPS and operando Raman and XAS spectroscopy. **a** High-resolution spectra of the Cu 2*p* regions and Cu L_3_M_45_M_45_ Auger transition modes measured by ex situ X-ray photoelectron spectroscopy (XPS) of pristine Ag–Cu sample (P), H_2_O_2_-treated Ag–Cu (H_2_O_2_-P), C_3_-, C_2_N-, N_3_N- and N_2_SN- functionalized Ag–Cu electrodes. The different colored shading areas represent the peaks of Cu 2*p*_1/2_(blue), Cu 2*p*_3/2_(light purple), ^1^G (pink) and ^3^F (light green), respectively. **b** Ex situ and operando Copper K-edge X-ray absorption near-edge structure (XANES) spectra of pristine and functionalized Ag–Cu electrodes. Inset: Average oxidation state of copper for the corresponding electrodes. **c** Operando Cu K-edge XANES spectra of N_2_SN- functionalized Ag–Cu electrode during CO_2_RR. The measurements were performed after holding the applied potential for 30 min. **d** Evolution of the Faradaic efficiency for C_2+_ and H_2_ measured at −1.2 V vs. RHE with the oxidation state of Cu. **e** Operando Raman spectra for pristine, C_3_-, C_2_N-, N_3_N- and N_2_SN-, functionalized Ag–Cu during CO_2_RR at a fixed potential of −1.2 V vs. RHE. The different colored shading areas represent the peaks of 280 cm^−1^ (light green), ~365 cm^−1^ (pink), and ~2000 cm^−1^ (blue), respectively. The spectra for all the other potentials are presented in Supplementary Fig. 26. **f** Relationship between the FE for C_2+_ products and the Raman peak areas of the frustrated rotational mode of CO at 280 cm^−1^, the Cu–CO stretch at 365 cm^−1,^ and the C≡O stretch at 1900–2120 cm^−1^, respectively. **g** Relationship between the FE for C_2+_ molecules and the ratio of CO_atop_ and CO_bridge_ on different Ag–Cu electrodes. The ratio was obtained from the integrated areas of the deconvoluted peaks of the Raman spectra (Supplementary Fig. 27). The error bars in **b**, **d**, **f**, and **g** correspond to the standard deviation of three independent measurements.

Next, we sought to explain the fundamental mechanism responsible for the improved CO_2_RR properties using ex situ X-ray photoelectron spectroscopy (XPS) and operando XAS. XPS was firstly used to characterize the surface composition and determine the oxidation state of Cu. From the Cu2*p* region, no significant change of the oxidation state of Cu can be detected from the functionalized catalysts (Fig. 3a, left). For comparison, after exposure to H_2_O_2_, the electrodes are clearly oxidized as confirmed by the apparition of Cu2*p*_3/2_ signals at binding energy at 934.6 eV and the satellite peak at 942.6 eV, which is attributed to the formation of Cu^2+^^48^. Our XPS results confirm that functionalization does not lead to a dramatic modification of the oxidation state of the surface of the Cu since there were no evident oxidation peaks in Cu2*p*. It is well-known that the small change of binding energy between Cu^1+^ and Cu^0^ makes the precise identification of Cu^1+^ impossible from the Cu2*p* regions^22^. To overcome this limitation, we, therefore, used the Cu Auger L_3_M_45_M_45_ transition to qualitatively discuss the presence of Cu^1+^ in functionalized Ag–Cu as this mode is known to be more sensitive to the modification of the electron density on the d-band of the metals^49,50^. It is well-known that the formation of Cu Auger L_3_M_45_M_45_ transition comes from the *L*_3_ (2*p*_3/2_) core-hole decay during the Auger process, in which two M_45_ (3*d*) electrons are responsible for the formation of a final 3*d*^8^ configuration of Cu^51–54^. The right panel of Fig. 3a presents the two final-state terms splitting from L–S coupling ^1^G and ^3^F, whose peak energy positions provide information on the valence configuration of Cu^22,51^. According to the previous investigations, the peak energy positions of ^1^G for the different oxidation states copper are detected at 917.1, 915.8, and 918.0 eV for CuO, Cu_2_O, and Cu, respectively^51–53^. Such differences are mainly due to the modification of the 3*d* and O2*p* electron configurations^54^. Compared with Cu^0^, the ^1^G peak in copper oxide is downshifted in energy and presents a broader shape, while the ^3^F peak is solely visible in the case of Cu^0^ ^22,55^. For pristine and C_3_- and C_2_N- functionalized Ag–Cu, we observed that the energy positions of the ^1^G peak are located at 918.3 eV(pristine), 915.9 eV (C_3_ and C_2_N), respectively, while the distinct ^3^F peak is detected at 918.2 eV for both C_3_- and C_2_N-Ag–Cu, in agreement with the existence of Cu^0^ (Supplementary Table 4). Conversely, in the case of the N_2_SN- and N_3_N samples, the ^1^G peak is identified at 915.8 and 916.0 eV, respectively, which is lower than that for Cu^0^ and Cu^2+^ and close to that of Cu^1+^ (915.8 eV). We also note that the ^3^F peak is also visible for both samples pointing out the presence of Cu^0^. These results indicate that the valence state of the N_2_SN and N_3_N samples may be Cu^δ+^ with 0 < δ < 1.

To precisely evaluate the electronic states of copper on functionalized Ag–Cu electrodes and eliminate the air effect on the electrode, we then performed in situ X-ray absorption near-edge spectroscopy (XANES). The absorption edges of functionalized catalysts reside between those of copper metal (Cu^0^) and Cu_2_O (Cu^1+^) used as references (Fig. 3b). To better compare the influence of the different functional groups, we estimated the copper oxidation state as a function of copper K-edge energy shift (Fig. 3b). The oxidation state of copper in the N_2_SN- and N_3_N- functionalized Ag–Cu was found to be +0.53 and +0.47 respectively—pointing out the withdrawing properties of the selected heterocycles (Supplementary Table 5). Remarkably, C_3_- and C_2_N-functionalized samples displayed a minimal shift by comparing with pristine Ag–Cu electrode and the Cu reference, suggesting the alkyl groups are not prone to modulate the oxidation state nor the coordination environment of Cu. To explore the stability of the electron-withdrawing ability of the grafted heterocycles, we measured the oxidation state of Cu post CO_2_RR using in situ XANES. After 30 min of operation at −1.2 V vs. RHE in the testing cell, the oxidation state of copper was estimated be +0.51 (inset Figs. 3b, c). This value is similar to that obtained from the freshly prepared samples: +0.53, which demonstrates the stability of the oxidation state of the functionalized Ag–Cu electrodes. Similarly, no obvious shift of the Cu K-edge was observed from the in situ XANES measurements at increasing applied potential up to −1.2 V vs. RHE and the spectra virtually overlap. This confirms the robustness of the oxidation state of the Cu thanks to the stable attachment of the functional groups (Fig. 3c and Supplementary Fig. 23). To better understand the role of Cu^δ+^ on the CO_2_RR properties, we investigated the influence of the copper oxidation state on the FE for C_2+_ and H_2_ (Fig. 3d). Remarkably, we identified a strong correlation between the oxidation state and the FE for C_2+_, which points out that the larger oxidation state of Cu benefits the CO_2_RR properties and the formation of C_2+_ products in line with recent findings from the literature^51,56^. To finally exclude any hydrophobicity effect on the enhanced selectivity for formation of C_2+_ products, we sought to prepare functionalized electrodes with similar water contact angles as for pristine Cu counterparts. We identified 1,3,4-thiadiazole-2,5-dithiol, N_2_SS, that shares the same thiadiazole structure but exhibits a water contact angle of 81° compared to 83.9° for pristine non-functionalized Cu. In H-cell configuration, the Faradaic efficiency for the formation of C_2+_ molecules on N_2_SS-Ag–Cu reaches 43.7% at −1.2 V vs. RHE compared to only 18.3% for Ag–Cu (Supplementary Fig. 24). To further demonstrate that the water contact angle has limited influence on the improved C_2+_ selectivity, we plotted the Faradaic efficiency as a function of the water contact angle. No relationship is clearly observed, emphasizing that the origin of the improved selectivity for C_2+_ is not primarily due to the surface properties of the Cu electrodes but rather the electron-withdrawing nature of the aromatic heterocycles as evidenced by our operando X-ray absorption spectroscopy measurements (Supplementary Figs. 6, 24 and 25).

**Fig. 4:**
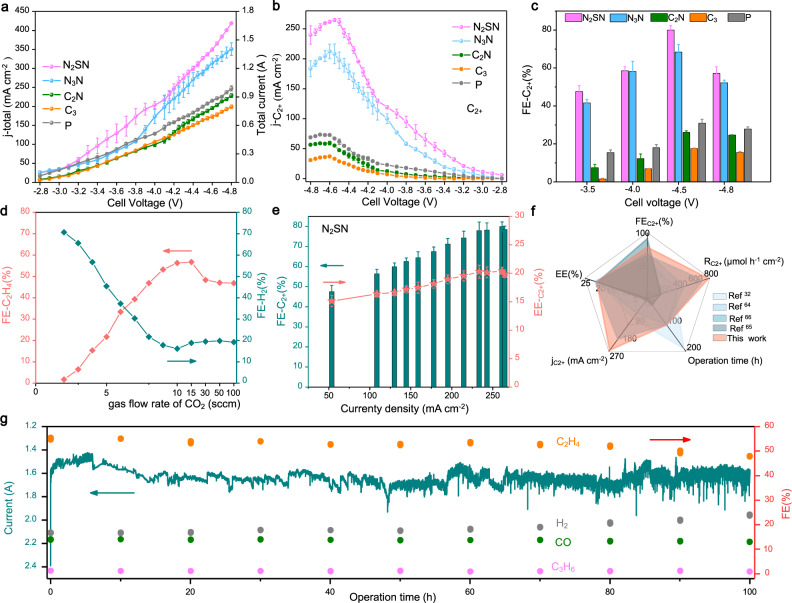
CO_2_RR performance of the functionalized Ag–Cu electrodes measured in MEA electrolyzers. **a** Relationship between the current and cell voltage relationship of pristine, C_3_-, C_2_N-, N_3_N- and N_2_SN- functionalized electrodes. The CO_2_RR electrolysis was operated using CO_2_ with a flow rate of 10 sccm, 0.1 M KHCO_3_ anolyte with a flow rate of 30 mL min^−1^. **b** Corresponding partial current density for the C_2+_ products. **c** Comparison of FEs for C_2+_ on the different Ag–Cu electrodes measured at full-cell potentials ranging between −3.5 and −4.8 V. **d** Evolution of the Faradaic efficiency for C_2+_ and H_2_ with the CO_2_ flow rate. **e** Evolution of the FEs and full-cell energy efficiency (EE) for C_2+_ as a function of specific current densities for C_2+_ on the N_2_SN- functionalized Ag–Cu electrode. The error bars represent the standard deviation of three independent samples measured under different current densities. **f** Comparison of the performance metrics of the MEA electrolyzers based on N_2_SN-functionalized Ag–Cu cathodes with literature benchmark. For each report, the plotted values are those corresponding to the longest duration test^32, 65–67^. The references are from refs. ^32^, ^65^, ^66^, and ^67^. **g** CO_2_RR performance of N_2_SN-Ag–Cu catalyst at a full-cell potential of −4.55 V and with a 10 sccm feed in CO_2_ over 100 h. The anolyte consisted in a 0.1 M KHCO_3_ solution with a flow rate of 30 ml min^−1^. The blue line represents the current recorded during the extended CO_2_RR experiment (primary *y* axis). Each orange, gray, green, and purple spheres represent the FEs for C_2_H_4_, H_2_, CO and C_3_H_6_ averaged from three independent measurements (secondary *y* axis). The error bars in **a**, **b**, **c**, and **e** represent the standard deviation of three independent measurements.

It is well-known that the formation of multicarbon products in CO_2_RR proceeds via the formation of the *CO intermediate, and its subsequent dimerization in CO=CO or *CO–COH intermediates^57–59^. To gain insight into the C–C coupling mechanism on functionalized and pristine Ag–Cu during CO_2_RR, the surface of the catalysts was probed using operando Raman spectroscopy in order to elucidate the interactions between the catalyst surface and the adsorbed *CO intermediate (Fig. 3e and Supplementary Fig. 26, and Supplementary Table 7). The presence of the surface-absorbed *CO was identified from the vibration modes at ≈280 and ≈365 cm^−1^ that originate from the Cu–CO frustrated rotation and Cu–CO stretch, respectively^60,61^. The broadband in the range of 1900–2120 cm^−1^ was assigned to the C≡O stretch. To confirm that the detected signals are solely due to the CO_2_RR, the Raman spectra were also recorded using Ar-saturated K_2_SO_4_ as a controlled experiment and no peaks were detected at these frequencies (Supplementary Fig. 26f). The Raman vibration modes around 1900–2120 cm^−1^ have recently been the focus of several studies and there is currently a general agreement that the high frequency region (>2000 cm^−1^) and the low-frequency region (1900–2000 cm^−1^) originates to atop-bound CO and bridge-bound CO. Atop (CO_top_) and bridge (CO_bridge_) configurations correspond to a CO bound on top of one Cu atom and between two Cu atoms, respectively^50,62,63^. Compared to pristine as well as 1-propanthiol- and cysteamine-functionalized electrodes, N_2_SN- and N_3_N-functionalized Ag–Cu exhibit the relatively intense signals at 365 and 1900–2000 cm^−1^. Our systematic investigations revealed that the intensities of both regions are also found to increase with the overpotentials^32^ (Supplementary Fig. 26a, b). Importantly, we observed that there is an obvious relationship between the peaks at 365 cm^−1^ and 1900–2100 cm^−1^ and the Faradaic efficiency towards the formation of C_2+_ products (Fig. 3f) by following literatures to fit these peaks area^32,50^. These results, therefore, point out the strong correlation between the density of adsorbed *CO on the catalyst surface and the formation of C–C bonds in agreement with the *CO being the key intermediate involved in the dimerization reaction and the formation of C_2+_ products. We note that 1-propanethiol functionalized Ag–Cu electrodes display the most intense peak at 280 cm^−1^ whereas no peak is detected at 1900–2120 cm^−1^. This indicates the adsorbed *CO is not present in the form of CO_atop_ nor CO_bridge_ configurations. We speculate that the hydrophobic surface of the 1-propanethiol functionalized Ag–Cu induces the existence of a high energy barrier for the protons to reach the surface of the catalyst that prevents the stabilization of the *CO in these bound configurations as previously proposed for other transition metals^50^. Interestingly, we observed a volcano-shaped relationship between the Faradaic efficiency for C_2+_ products and the ratio of atop-bound CO to bridge-bound CO on the surface of Ag–Cu (Fig. 4g and Supplementary Fig. 27). The Faradaic efficiency reaches a maximum for a ratio of CO_atop_ to CO_bridge_ of 0.4–0.5 corresponding to thiadiazole and triazole-functionalized catalysts, while the ratio decreases for 1-propanethiol and increases for pristine and cysteamine respectively. We hypothesized that the density of CO_atop_ and CO_bridge_ on the surface of the catalysts is influenced by the electron-withdrawing ability of the heterocycles as suggested by the volcano-shaped relationship between the oxidation state of Cu and the ratio of CO_atop_ to CO_bridge_ (Supplementary Fig. 28). Overall our ex situ and operando characterizations of the modified bimetallic catalyst establish an obvious correlation between the electron-withdrawing ability of the functional groups and the oxidation state of Cu, which translate into a larger concentration of adsorbed *CO on the electrode surface and ultimately a higher probability for *CO to dimerize.

### CO_2_RR using a membrane electrode-assembly (MEA) flow electrolyzer

To evaluate the potential of our approach for practical applications towards the electrosynthesis of C_2+_ products, we integrated the different functionalized bimetallic electrodes into 4 cm^2^ membrane electrode-assembly (MEA) flow electrolyzers (Supplementary Fig. 29). The synthesized liquid products at the cathode were collected by using a cold trap connected to the cathode gas outlet. We also analyzed the liquid products in the anolyte to detect liquid products that may have crossed over the membrane electrolyte. We firstly scrutinized the activity of N_2_SN-functionalized Ag–Cu in a MEA electrolyzer by flowing Ar (used as a blank experiment) and CO_2_ in the cathode compartment (Supplementary Fig. 30) and found that the catalyst can convert CO_2_ when operating in a catholyte-free MEA system. We then characterized the current-voltage response of all the functionalized catalysts between −2.8 and −4.8 V and a constant flow of CO_2_ of 10 standard cubic centimeters per minute (sccm) (Fig. 4a). The total current for the different Ag–Cu electrodes increased from 4·10^−2^ A up to over 1.6 A. The N_2_SN-functionalized electrodes displayed the largest specific current density for C_2+_ at 261 mA cm^−2^ together with the maximum FE for C_2+_ products and the lowest FE for H_2_ at ~80% and 14%, respectively (Fig. 4b and Supplementary Figs. 31a, 32a). Remarkably the selectivity for the C_2+_ products increases together with the electrolysis response when increasing the operating potential of the full cell. The catalytic activity towards the competitive HER concurrently decreases up to −4.55 V (Fig. 4b and Supplementary Fig. 32c). Compared to pristine Ag–Cu, the FE for C_2+_ products from N_2_SN- and N_3_N-functionalized electrodes demonstrated an average enhancement for C_2+_ of 3.1 and 2.6 folds respectively over the extended range of full-cell potentials (Fig. 4c and Supplementary Fig. 33). To further assess the performance of the functionalized Ag–Cu electrodes in the MEA devices, we calculated the ratio of ${{{{{{\rm{j}}}}}}}_{{{{{{{\rm{C}}}}}}}_{2+}}$ to ${{{{{{\rm{j}}}}}}}_{{{{{{{\rm{C}}}}}}}_{1}}$ for the different potentials. We found that Ag–Cu functionalized with thiadiazole displays the largest values and the ratio reaches at a maximum value of ≈10 at a current density of 261.4 mA cm^−2^ (Supplementary Fig. 34). These results demonstrate that the controlled orientation of the reaction pathways towards the synthesis of ethanol and ethylene observed in the H-cell reactors can be transposed to the MEA devices (Supplementary Fig. 29). We also found that the total FE for gaseous products gradually decreased with the increase of the full-cell voltage indicating a shift toward the formation of liquid products at high operating potential. The Faradaic efficiency for ethanol and n-propanol reached 16.5% and 6.1% at a voltage of −4.4 V (Supplementary Fig. 31a).

To better understand the influence of operating conditions on the CO_2_RR performance of the MEA device, we varied the CO_2_ flow rate from 3 to 100 sccm at a constant full-cell potential of −4.55 V. When using N_2_SN-functionalized Ag–Cu electrodes, the FE for ethylene reached a peak at 56% at ~10 sccm (Fig. 4d) together with a sharply reduced FE for H_2_ at only 15.2%. The selectivity for ethylene rapidly drops down to only ~5% for a CO_2_ flow rate of 3 sccm, suggesting that the feed in CO_2_ is not sufficient to produce enough *CO to dimerize on the surface of the catalyst. The relationships between CO_2_ flow rates, cell voltages and Faradaic efficiencies for the main gas products (H_2_, CO and C_2_H_4_) were explored on N_2_SN-functionalized Ag–Cu electrodes and we found that the FE_C2H4_ decreases when increasing the CO_2_ flow rate and the optimal flow rate is 10 sccm even when operating under high voltage and high current density (Supplementary Fig. 35). Conversely, the Faradaic efficiency for H_2_ increases when increasing the CO_2_ flow rate, which further demonstrates that the decrease in the C_2+_ performance is not caused by insufficient feed in CO_2_. We also estimated the full-cell energy efficiency (EE_full-cell_) for N_2_SN-functionalized Ag–Cu for the different operating potential. Both the FE and EE_full-cell_ values for C_2+_ products increased with the increase of the current density and achieved a maximum FE_C2+_ of ≈80 ± 1% and an EE_full-cell_ of 20.3% at a specific current density larger than 260 mA cm^−2^ for the production of C_2+_ (Fig. 4e). By comparing the performance metrics of N_2_SN-functionalized Ag–Cu with previous literature benchmarks based on MEA devices, we observed that thiadiazole-functionalized Ag–Cu allows achieving outstanding performance notably thanks to a high CO_2_-to-C_2+_ conversion rate of 785 µmol h^−1^ cm^−2^ (Fig. 4f).

We finally examined the stability of the N_2_SN-functionzalized Ag–Cu electrodes in a full-cell MEA electrolyzer under continuous operation at a CO_2_ flow rate of 10 sccm and a cell voltage of −4.55 V. The performance of the cell was found to be stable over 100 h with an average FE of 51% for ethylene and an average current of around 1.6 A (Fig. 4g). After 100 h, the retention of the FE for ethylene and the current were estimated to be 48% and 1.58 A corresponding to retentions of 94% and 99%, respectively. The stability of the CO_2_RR properties is further accompanied by a high stability of the catalyst morphology and microstructure (Supplementary Fig. 36).

## Discussion

Our study describes an original and robust molecular engineering strategy to tune the oxidation state of Cu electrodes via functionalization. We identified that strong electron-withdrawing groups based on aromatic heterocycles can effectively orient the pathway of the CO_2_RR reactions towards the synthesis of C_2+_ molecules. Functionalization of the surface of a bimetallic Ag–Cu catalyst with thiadiazole and triazole derivatives led to an enhancement of the ${{{{{\rm{F}}}}}}{{{{{{\rm{E}}}}}}}_{{{{{{{\rm{C}}}}}}}_{2+}}$ up to ≈80 ± 1%, corresponding to ratios of ${{{{{\rm{F}}}}}}{{{{{{\rm{E}}}}}}}_{{{{{{{\rm{C}}}}}}}_{2+}}$ to ${{{{{\rm{F}}}}}}{{{{{{\rm{E}}}}}}}_{{{{{{{\rm{C}}}}}}}_{1}}$ and ${{{{{\rm{F}}}}}}{{{{{{\rm{E}}}}}}}_{{{{{{{\rm{C}}}}}}}_{2+}}$ to ${{{{{\rm{F}}}}}}{{{{{{\rm{E}}}}}}}_{{{{{{{\rm{H}}}}}}}_{2}}$ of 10 and 5.3, respectively. By combining Auger and XANES spectroscopy we identified that the superior performance towards the CO_2_-to-C_2+_ conversion originates from the controlled p-doping of the Cu and presence of Cu^δ+^ with 0 < δ < 1. The functionalized Ag–Cu electrodes were found stable, which translates into a prolonged production of C_2+_ products for >100 h.

## Methods

### Chemicals

Copper sulfates (CuSO_4_, 99%), silver nitrate (AgNO_3_, 99%), ammonium sulfate(99%, ethylenediamine(NH_2_CH_2_CH_2_NH_2_, 99.5%), potassium hydroxide (KOH, 90%) potassium bicarbonate(KHCO_3_, 99.7%), sulfuric acid(H_2_SO_4_, 99.99%), Iridium (III) chloride hydrate (IrCl_3_·*x*H_2_O, 99.9%), 5-Amino-1,3,4-thiadiazole-2-thiol(C_2_H_3_N_3_S_2_,95%), 3-amino-1,2,4-triazole-5-thiol(C_2_H_4_N_4_S,99%), cysteamine(C_2_H_7_NS,99%) and 1-Propanethiol (C_3_H_8_S, 99%) were purchased from Sigma Aldrich. Nafion 117 and anion exchange membrane (Fumapem FAA-3-50), gas diffusion layer (Freudenberg, H23C6), and titanium mesh were obtained from Fuel Cell Store. All chemicals were used as received. All aqueous solutions were prepared using deionized water with a resistivity of 18.2 MΩ cm^−1^.

### Electrodes preparation

Before depositing catalysts, gas diffusion electrode (GDE) was treated with sulfuric acid by sonicating 20 min. After acid treatment, the remaining acid was rinsed with deionized water for 5 min three times, and gas diffusion layer was dried at room temperature. To obtain the working electrodes, 15%_at._ Ag–Cu catalysts were prepared through a pulse electrodeposition approach under CO_2_ bubbling condition. Firstly, electrochemical deposition of the Ag catalyst was performed using a potentiostat (VSP potentiostat from Bio-Logic Science Instruments). The electrolyte used was composed of 0.01 M AgNO_3_, 0.6 M (NH_4_)_2_SO_4_, and 0.04 M ethylenediamine. Ag catalyst was electrodeposited on GDE at a current density of 15 mA cm^−2^ with on- and off-time pulsing parameters of 0.25 and 3 s, respectively. Then, the Cu was electrodeposited on Ag at a constant current density of −400 mA cm^−2^ for 45 s to obtain the 15%_at._ Ag–Cu electrode. The solution consisted of 0.2 M CuSO_4_ and 1 M H_2_SO_4_ with continuous CO_2_ bubbling.

### Functionalization of the Ag–Cu electrodes

The different functional groups (organic chemicals(5-Amino-1,3,4-thiadiazole-2-thiol(N_2_SN), 1,3,4-thiadiazole-2,5-dithiol (N_2_SS), 3-amino-1,2,4-triazole-5-thiol(N_3_N), cysteamine(C_2_N) and 1-Propanethiol (C_3_)) were dissolved in ethanol to a fixed concentration of 5 mM. The Ag–Cu electrodes were treated by the different functional solutions via drop-casting 20 μL of the solution containing the different thiol reagents on the GDE. After 5 min, the electrode was washed with ethanol and dried under argon flow.

### Physical characterizations

A field emission scanning electron microscope (TESCAN Mira3) was employed to observe the morphology of samples. Aberration-corrected high-resolution (scanning) TEM imaging (HR-(S)TEM), energy-dispersive X-ray spectroscopy (EDS) and spatially-resolved electron energy-loss spectroscopy (SR-EELS) were performed using a FEI Titan Cubed Themis microscope which was operated at 80 kV. The Themis is equipped with a double Cs aberration corrector, a monochromator, an X-FEG gun, a super EDS detector, and an Ultra High Resolution Energy Filter (Gatan Quantum ERS) which allows for working in Dual-EELS mode. HR-STEM imaging was performed by using high-angle annular dark-field (HAADF) and annular dark-field (ADF) detectors. SR-EELS spectra were acquired with the monochromator excited allowing an energy resolution of 1.1 eV with an energy dispersion of 0.4 eV pixel^−1^. Liquid products were quantified by 1H NMR spectroscopy (600 MHz Avance III Bukrer with a cryorobe Prodigy TCI) using deionized water with 0.1% (w/w) of DSS (Sodium trimethylsilyl propane sulfonate) like internal standard for the quantification of the ethanol and formate. An 1D sequence water suppression with excitation sculpting with gradients (zgesgp) was used for the acquisition (Number of scan = 32, Delay D1 = 30 s). X-ray photoelectron spectroscopy (XPS) measurements were carried out on Thermo Electron ESCALAB 250 System using Al Kα X-ray radiation (1486.6 eV) for excitation. Raman measurements were conducted using a Renishaw in Via Raman microscope and an ×50 objective (Leica) equipped with a 633 nm laser. Operando Raman measurements were carried out using a modified liquid-electrolyte flow cell. The spectra were recorded and processed using the Renishaw WiRE software (version 4.4). An Ag/AgCl electrode and a Pt plate were used as the reference and counter electrodes respectively. Ex situ X-ray absorption spectra at the copper K-edges and Operando X-ray absorption spectroscopy (XAS) measurements at the copper K-edges were collected at Beijing Synchrotron Radiation Facility (BSRF) on beamline 1W1B and the SOLEIL synchrotron SAMBA beamline, respectively.

### Operando X-ray absorption spectroscopy (XAS)

Operando XAS measurements at the copper K-edges were collected at Beijing Synchrotron Radiation Facility (BSRF) on beamline 1W1B and the SOLEIL synchrotron SAMBA beamline, respectively. Operando XAS measurements of functionalized Ag–Cu electrodes were obtained by using a Si (111) monochromator at the Cu K-edge for energy selection with the beam size of 1 × 0.5 mm. A 13-channel Ge detector was used to collect the signals in fluorescence mode. An ionization chamber (*I*_0_) filled with a mixture of N_2_/He was used to measure the intensity of the incident radiation. While the measurements in transmission mode were operated in other ionization chambers which were filled with the mixture of N_2_ and Ar in I_1_ chamber. A modified electrochemical cell was used for operando XAS measurements. The applied potential was controlled by a VSP potentiostat (Bio-Logic Science Instruments). A platinum wire and Ag/AgCl electrode (3 M KCl) were used as counter and reference electrodes, respectively. For the XAS studies, 15%at. Ag–Cu was firstly electrodeposited on gas diffusion layer (GDL, Sigracet 22 BB, Fuel Cell Store) used as gas diffusion electrode (GDE) and then functional solutions were drop-coated on the catalyst side, while the other side of the GDL was covered with polyamide tape. The GDL was then tape on a graphite foil and subsequently, the electrode was mounted in an operando cell with the graphite foil acting as a working electrode and window. A 0.5 M solution of KHCO_3_ was used as electrolyte for the CO_2_RR and the cell was continuously purged with CO_2_ during the measurements. All measurements were performed at constant potentials of −1.2 V, −1.1 V, −1.0 V and −0.9 V vs. RHE. Time-resolved spectra were recorded every 30 min until no further changes were observed under CO_2_RR conditions.

Data alignment and normalization of the X-ray absorption near-edge structure (XANES) spectra were conducted by using the Athena software. To fit the Cu K-edge extended X-ray absorption fine structure (EXAFS) spectra χ(*k*)*k*^2^, the range of parameter *R* in R-space from *R*_min_ = 1 Å up to *R*_max_ = 2.1 Å was used for the freshly prepared catalysts, while *R*_min_ = 1.0 Å to *R*_max_ = 3.0 Å were used for the reductive catalysts. The *k*-range from 3.0 Å^−1^ to 10.0 Å^−1^ with a *k*-weighting of 1, 2, and 3 were applied in the Fourier transforms. All fitting parameters including the coordination numbers *N*, interatomic distances *R*, disorder factors *σ*^2^ for Cu–O and Cu–Cu paths, as well as the corrections to the photoelectron reference energies Δ*E*_0_ were obtained. The *S*_0_^2^ factors were set to 0.831.

### Computational details

All density functional theory (DFT) calculations were carried out in the Vienna Ab-initio Simulation Package (VASP) code with the projector augmented-wave (PAW) method. The exchange–correlation energy was treated using a general gradient approximation (GGA) with the Perdew–Burke–Ernzerhof (PBE) formalism. A plane-wave basis with a kinetic energy cutoff of 500 eV was chosen to expand the electronic wave functions. To investigate the possible binding modes between functional molecular and catalysts, a 5 layers of Cu (111) slab (7.7386 Å × 7.7386 Å), in which the two bottom layers were kept fixed during relaxation, was built with a vacuum space of about 20 Å. For the geometrical optimizations, all atoms were fully relaxed to the ground state with the convergence of energy and forces setting to 1.0 × 10^−5 ^eV and 0.01 eV Å^−1^, where a 3 × 3 × 1 Γ-centered Monkhorst-Pack schemed *k*-mesh was used to sample the first Brillouin zone. To compare the bond strength between each group of functional molecular and Cu (111), the adsorption energy (*E*_ads_) is calculated by using the following formula:

1 $${E}_{{{{{{{\mathrm{ads}}}}}}}}={E}_{{{{{{{\mathrm{Cu}}}}}}}/{{{{{{\mathrm{FM}}}}}}}}-{E}_{{{{{{{\mathrm{Cu}}}}}}}}-{E}_{{{{{{{\mathrm{FM}}}}}}}}$$

where *E*_Cu/FM_, *E*_Cu_, and *E*_FM_ denote the total electronic energies of an adsorbed system, a clean Cu (111) surface, and the free functional molecular, respectively.

### Electrochemical in H-cell and MEA configuration

All electrochemical measurements were carried out at an ambient temperature and pressure using a VSP electrochemical station from Bio-Logic Science Instruments equipped with a 5 A booster and FRA32 module. The cell voltages reported in all figures were recorded without iR correction. All the potentials in the H-cell were converted to values with reference to the RHE using:

2 $${{{E}}}_{{{{{{\rm{RHE}}}}}}}={{{E}}}_{{{{{{\rm{Ag}}}}}}/{{{{{\rm{AgCl}}}}}}}+0.197{{{{{\rm{V}}}}}}+0.0591\times {{{{{\rm{pH}}}}}}$$

In the H-cell configuration, Ag/AgCl reference electrode (3 M KCl) and Pt plate were used as a reference and counter electrodes, respectively. The electrolyte consisted of a 0.5 M KHCO_3_ solution (99.9%, Sigma Aldrich), which was saturated with alternatively CO_2_ (≥99.998, Linde) or Ar (5.0, Linde). Prior to any experiment, the electrolyte solutions were saturated by bubbling CO_2_ or Ar for at least 20 min.

The electrochemically active surface area (ECSA) of the different catalysts was determined using Pb underpotential deposition in H-cell. An Ar-saturated solution of 100 mM HClO_4_ + 1 mM Pb(ClO_4_)_2_ was used as an electrolyte. The working electrode was held at −0.7 V vs. Ag/AgCl for 10 min and then cyclic voltammetry was recorded between −0.7 and 0.7 V vs. Ag/AgCl at 10 mV s^−1^. Pt foil was used as the counter electrode, while Ar (Linde, 99.998%) was continuously supplied to the electrolyte. The ECSA values for Cu and Ag were calculated assuming the deposition of a monolayer of Pb atoms over Cu and Ag surface with a conversion factor of 310 and 260 mC cm^−2^, respectively^64^.

The MEA electrolyzer (Dioxide Materials) was comprised of the Ag–Cu cathode, a Ti-IrO_*x*_ mesh anode, and an anion exchange membrane (AEM, Fumasep FAA-3-50, Fuel cell store). The anode and cathode flow fields are made of titanium and stainless steel with geometric active areas of 4 cm^2^, respectively. The anode was prepared by following previous work through depositing IrO_*x*_ on a titanium support (0.002″ thickness, Fuel Cell Store) with a loading of 2 mg cm^−2^ by using a dip coating method followed by thermal annealing^65^.

The MEA was prepared by hot-pressing the anion exchange membrane (AEM, Fumapem FAA-3-50, Dioxide Materials) between the Ag–Cu cathode and Ti-IrO_*x*_ anode. The cell was assembled with flow fields for the anode and the cathode, which were separated by the AEM. Anode and cathode gaskets were used to ensure good sealing of the electrolyzer (Supplementary Fig. 29). A 0.1 M KHCO_3_ anolyte and humidified CO_2_ gas were fed to the anode and cathode at constant flow rates of 30 mL min^−1^ and 10 standard cubic centimeters per minute (sccm), respectively. The voltage of the electrolyzer was progressively increased from −2.8 V with increments of 50 or 100 mV. After 15–20 min of stable operation under constant full-cell potentials, the products were collected and analyzed.

### Quantification of the CO_2_RR products

The electrochemical data were recorded while simultaneously collecting the CO_2_RR gas products by using an automatic sampler connected to the cathode outlet. A cold trap was used to collect the liquid products before the sampler. For each applied potential, the gas products were collected at least three times with proper time intervals. The gas aliquots were then injected into an online gas chromatograph (Agilent, Micro GC-490) equipped with a TCD detector and Molsieve 5 A column continuously. Hydrogen and argon (99.9999%) were used as the carrier gases. Liquid products were quantified by 1H NMR spectroscopy (600 Mhz Avance III Bukrer with a cryorobe Prodigy TCI) using deionized water with 0.1% (w/w) of DSS (Sodium trimethylsilylpropanesulfonate) like internal standard for the quantification of the ethanol and formate. An 1D sequence water suppression with excitation sculpting with gradients(zgesgp) was used for the acquisition (Number of scan = 32, Delay D1 = 30 s). Owing to the liquid product crossover, the FE values of the liquid products were calculated based on the total amount of the products collected on the anode and cathode sides during the same period.

### Stability measurements in the MEA configuration

For the stability test, the MEA electrolyzer was operated at a constant voltage of −4.55 V with continuous feeding in CO_2_. The gas products were collected at frequent time intervals. The FE values were calculated from the average value obtained from three successive injections. As for the liquid products, the total liquid products were collected at the end of the experiments.

### Faradaic efficiency and energy efficiency calculations

The Faradaic efficiency (FE) of each gas product was calculated as follows:

3 $${{{{{{\rm{FE}}}}}}}_{{{{{{\rm{gas}}}}}}}={{{g}}}_{{{i}}}\times {{v}}\times \frac{{{{z}}}_{{{i}}}}{{{RT}}}{{{{{\rm{F}}}}}}{{{{{{\rm{P}}}}}}}_{0}\times \frac{1}{{{{I}}}_{{{{{{\rm{total}}}}}}}}\times 100 \%$$

The Faradaic efficiency (FE) of each liquid product was calculated as follows:

4 $${{{{{{\rm{FE}}}}}}}_{{{{{{\rm{liquid}}}}}}}={{{l}}}_{{{i}}}\times \frac{{{{z}}}_{{{i}}}}{{{{Q}}}_{{{{{{\rm{total}}}}}}}}{{F}}\times 100 \%$$

The formation rate (*R*) for each species (*i*) was calculated as follows:

5 $${{{R}}}_{{{i}}}=\frac{{{{Q}}}_{{{{{{\rm{total}}}}}}}\times {{{{{{\rm{FE}}}}}}}_{{{i}}}}{96,485\times {{{z}}}_{{{i}}}\times {{t}}\times {{S}}}$$

The full-cell energy efficiencies (EE) was calculated as follows:

6 $${{{{{\rm{EE}}}}}}=\frac{(1.23-{{{E}}}_{{{i}}})\times {{{{{\rm{F}}}}}}{{{{{{\rm{E}}}}}}}_{{{i}}}}{{{{E}}}_{{{{{{\rm{cell}}}}}}}}$$

where *g*_*i*_ represents the volume fraction of gas product *i*; *v* represents the gas flow rate at the outlet in sccm; *z*_*i*_ represents the number of electrons required to produce one molecule of product *i*; *I*_total_ represents the total current; *l*_*i*_ represents the number of moles of liquid product *i*; and *Q*_total_ represents the charge passed while the liquid products are being collected. *P*_0_ = 1.01 × 10^5^ Pa, *T* = 273.15 K, *F* = 96,485 C mol^−1^ and *R* = 8.314 J mol^−1^ K^−1^; *t* represents the electrolysis time (*h*); *S* represents the geometric area of the electrode (cm^2^); *E*_*i*_ represents the thermodynamic potential (versus RHE) for CO_2_RR to species *i* and *E*_cell_ represents the cell voltage in two-electrode setup.

## Supplementary information

Supporting Information

## Data Availability

The authors declare that all data supporting the results of this study are available within the paper and its Supplementary Information files or from the corresponding authors upon reasonable request.
