# Supplementary material for: Improved electrochemical conversion of CO2 to multicarbon products by using molecular doping
Source: Nat Commun. 2021 Dec 10;12:7210. doi: 10.1038/s41467-021-27456-5 (PMC8664807; doi:10.1038/s41467-021-27456-5)
Supplement: Supplementary file 1 — Supporting Information [file 41467_2021_27456_MOESM1_ESM.pdf]

**Supplementary Information for**

# Improved electrochemical conversion of $CO_2$ to multicarbon products by using molecular doping

Huali Wu[1], Ji Li[1,2], Kun Qi[1], Yang Zhang[1], Eddy Petit[1], Wensen Wang[1], Valérie Flaud[3], Nicolas Onofrio[1], Bertrand Rebiere[3], Lingqi Huang[4], Chrystelle Salameh[1], Luc Lajaunie[5,6], Philippe Miele[1,7], Damien Voiry[1]*

*[1] Institut Européen des Membranes, IEM, UMR 5635, Université Montpellier, ENSCM, CNRS, Montpellier 34000, France*
*[2]College of Bioresources and Materials Engineering, Shaanxi University of Science & Technology, Xi'an 710021, PR China*
*[3] Institut Charles Gerhardt, ICGM, UMR 5253, University of Montpellier, ENSCM, CNRS, 34095 Montpellier Cedex5, France*
*[4]School of Science and Engineering, The Chinese University of Hong Kong, Shenzhen, Guangdong, 518172, PR China*
*[5] Departamento de Ciencia de los Materiales e Ingeniería Metalúrgica y Química Inorgánica, Facultad de Ciencias, Universidad de Cádiz, Campus Río San Pedro S/N, Puerto Real, 11510, Cádiz, Spain*
*[6] Instituto Universitario de Investigación de Microscopía Electrónica y Materiales (IMEYMAT), Facultad de Ciencias, Universidad de Cádiz, Campus Río San Pedro S/N, Puerto Real 11510, Cádiz, Spain*
*[7] Institut Universitaire de France (IUF), 1 rue Descartes, 75231 Paris Cedex 05*

**Table of content**

## Sections

Improved electrochemical conversion of $CO_2$ to multicarbon products by using molecular doping ........ 1

Supplementary Fig.1 SEM images of pure Ag........ 8

Supplementary Fig. 2 SEM images of the Ag-Cu electrodes with different Ag atomic ratios. SEM images of different atomic ratios of Ag in Ag-Cu electrodes（0%at., 10 %at., 15 %at., 25 %at. and 50 %at.）. ........ 9

Supplementary Fig. 3 Comparisons of the current density on the different catalysts measured in the H-cell reactors. The current density for the different Ag atomic ratios in Ag-Cu catalysts (0%at. AgCu (yellow), 10%at. AgCu (purple), 15%at. AgCu (gray), 25%at. AgCu (green), and 50%at. AgCu(red)). The error bars represent the standard deviation of three independent samples during the entire operation and for each cell voltage........ 10

Supplementary Fig. 4 Molecular structures of the different molecules used for the functionalization of Ag-Cu. ........ 11

Supplementary Fig. 5 SEM figures for the pristine and functionalized Ag-Cu. SEM figures of different functional groups modified 15 %at. Ag-Cu electrodes: P (a), (b) $N_3N$, (c) $C_2N$ and (d) $C_3$. ........ 12

Supplementary Fig. 6 Wettability of the pristine and functionalized Ag-Cu electrodes. The water contact angles measured for 15 %at. Ag-Cu: P (a), $N_2SN$ (b), $N_3N$ (c), $C_2N$ (d), $C_3$ (e) and $N_2SS$ (f) before $CO_2RR$. ........ 13

Supplementary Fig. 7 Structural and compositional analyses of the 15 %at. Ag-Cu-$N_2SN$ catalyst. Low magnification SEM images (top left panel) and the related EDX elemental mapping of Cu (green), Ag (purple), C (red), N (dark blue), and S(orange) (right panels)........ 14

Supplementary Fig. 8 HAADF-STEM data of $N_2SN$-functionalized Ag-Cu catalyst. *Right panel:* The superposition of the HAADF-STEM image of the $N_2SN$ functionalized Ag-Cu ultrathin section with the sulfur (S) EDS elemental map. The arrow highlights the area used to extract the intensity profiles. *Left panel:* The corresponding intensity profiles of the HAADF-STEM images and S elemental map. ........ 15

Supplementary Fig. 9 Fourier transformed infrared (FTIR) spectra of the pristine functional groups. The ATR-FTIR spectra of pristine (non-functionalized) 15 %at. Ag-Cu (gray), 15 %at. Ag-Cu-$N_2$SN (purple), 15 %at. Ag-Cu-$N_3$N (blue), 15 % at. Ag-Cu-$C_2$N (green) and 15 %at. Ag-Cu-$C_3$ (orange) before $CO_2$RR. ........................................16

Supplementary Fig. 10 X-ray photoelectron spectra (XPS) spectra of the different Ag-Cu catalysts. The high-resolution XPS spectra of the Ag3*d* (a), S2*p* (b) and N1*s* (c) regions of the different functionalized 15 %at. Ag-Cu catalysts before reaction. ........17

Supplementary Fig. 11 X-ray diffraction (XRD) data of the different Ag-Cu catalysts. Powder XRD spectra of 15 %at. Ag-Cu and 15 %at. Ag-Cu-$N_2$SN compared with Cu, Ag metals used as references. ..................................................................................18

Supplementary Fig. 12 Schematic representations of the different configurations of $N_2$SN on Cu (side view). Molecular structure of $N_2$SN (a), $S_1$-$C_2$-$S_2$ flat model (b), $S_1$ model (c), $N_2$-$N_3$ model (d), $N_1$-$S_1$ model (e) and $S_1$-$S_2$ model (f). ................................19

Supplementary Fig. 13 The different configurations of $N_2$SN on Cu (top view) used for the calculation results summarized in Supplementary Table 1. $S_1$-$C_2$-$S_2$ flat model (a), $S_1$ model (b), $N_2$-$N_3$ model (c), $N_1$-$S_1$ model (d) and $S_1$-$S_2$ model (e). ..........................20

Supplementary Fig. 14 Comparisons of the Faradaic efficiencies on the different catalysts measured in the H-cell reactors. The Faradaic efficiency for the different products on $N_2$SN-(a), $N_3$N-(b), $C_2$N-(d) and $C_3$-(e) Ag-Cu electrodes, as well as pristine sample (c). The error bars represent the standard deviation of three independent samples during the entire operation and for each cell voltage. .......................................21

Supplementary Fig.15 Representative NMR spectra of the liquid products formed on $N_2$SN-functionalized Ag-Cu. $^1$H NMR spectra of liquid products collected from the electrolyte after 30 min at −1.2 V versus the reversible hydrogen electrode (vs. RHE). ..............................................................................................................................22

Supplementary Fig. 16 $CO_2$RR performance in the H-cell reactors. a, b FE values for $C_1$ products (a) and $H_2$ (b) on the different catalysts at various potentials ranging from -0.3 to −1.4 V vs. RHE in 0.5 M $KHCO_3$. c, *j*–V plots of the total current densities versus the RHE on different samples in 0.5 M $KHCO_3$. The error bars represent the standard deviation of three independent measurements...................................................23

Supplementary Fig. 17 CVs for different samples measured in 100 mM $HClO_4$ + 1 mM $Pb(ClO_4)_2$. ...........................................................................................................................24

Supplementary Fig. 18 Partial $C_{2+}$ products current density normalized to Cu ECSA for 15at.% Ag-Cu(gray) and $N_2$SN-15at.% Ag-Cu (dark blue) catalysts versus potential

for $CO_2$RR in H-cell. The error bars represent the standard deviation of three independent measurements. ........ 25

Supplementary Fig. 19 Electrochemical impedance spectroscopy (EIS) measured for the different Ag-Cu catalysts. The EIS spectra measured in the H-cell configuration for pristine (non-functionalized) 15 %at. Ag-Cu (gray), 15 %at. Ag-Cu-$N_2$SN (purple), 15 %at. Ag-Cu-$N_3$N (blue), 15 % at. Ag-Cu-$C_2$N (green) and 15 % at. Ag-Cu-$C_3$ (orange). The EIS data were recorded in $CO_2$-saturated 0.5 M $KHCO_3$ solution. ........ 26

Supplementary Fig. 20 Stability measurements of $N_2$SN-, $N_3$N- functionalized Ag-Cu compared with pristine Ag-Cu measured in the H-cell reactors. The stability of Ag-Cu-$N_2$SN (pink), Ag-Cu-$N_3$N (blue) and Ag-Cu (gray) were obtained at -1.2 V vs. RHE without *iR*-correction. ........ 27

Supplementary Fig. 21 X-ray photoelectron spectra of $N_2$SN before and after $CO_2$RR. The XPS data from the Cu 2*p* (a), S 2*p* (b) and N1*s* (c) regions were measured before and after operation up to 100 h at -1.2 V vs. RHE. ........ 28

Supplementary Fig. 22 Estimated atomic N/Cu ratio of the $N_2$SN-Ag-Cu electrodes along the $CO_2$RR operating time. The atomic N/Cu ratio of $N_2$SN-Ag-Cu were estimated from the deconvoluted XPS spectra shown in Supplementary Figure 21. The error bars represent the standard deviation of three independent measurements. ........ 29

Supplementary Fig. 23 Cu K-edge X-ray absorption near edge structure (XANES) spectra of the different Ag-Cu catalysts. Magnification of the *operando* Cu K-edge XANES spectra of $N_2$SN-functionalized Ag-Cu electrode during $CO_2$RR. The operando XANES measurements were performed after applying a fixed potential vs. RHE for 30 minutes. ........ 30

Supplementary Fig. 24 The Faradaic efficiency for the different products on $N_2$SS functionalized 15at.% Ag-Cu catalyst. The error bars represent the standard deviation of three independent measurements. ........ 31

Supplementary Fig. 25 The relationship between water contact angle and Faradaic efficiency of $C_{2+}$ products in H-cell for 15at.% Ag-Cu(P), $N_2$SN, $N_3$N, $C_2$N, $C_3$ and $N_2$SS. The error bars represent the standard deviation of three independent measurements. ........ 32

Supplementary Fig. 26 Operando Raman spectra of (a) $N_2$SN-, (b) $N_3$N-, (c) $C_2$N-, and (d) $C_3$-functionalized Ag-Cu electrodes compared with (e) pristine Ag-Cu. a-e, The operando Raman measurements were carried out between -0.7 V and -1.2 V vs. RHE in a $CO_2$-saturated $KHCO_3$ solution. To confirm that the signals are solely coming from the $CO_2$RR, the $N_2$SN-functionalized Ag-Cu catalyst was also tested in an Ar-saturated

$K_2SO_4$ electrolyte solution (f). ........ 33

Supplementary Fig. 27 Deconvolution of the Raman signals around 2000 $cm^{-1}$. *Right panel:* Operando Raman spectra centered around 2000 $cm^{-1}$ representing the C≡O stretch region on pristine and functionalized Ag-Cu electrodes. The asymmetric signals were deconvoluted into two components for the atop and the bridge CO using Lorentzian curves. The ratio of the intensities (in peak area) of the two bands is summarized in Supplementary Table 7. *Left panel:* Schematic representation and photograph of the operando setup. ........ 34

Supplementary Fig. 28 Relationship between the ratio of $CO_{atop}$ and $CO_{bridge}$ with the oxidation degree of Cu obtained from our XANES measurements. The error bars represent the standard deviation of three independent measurements. ........ 35

Supplementary Fig. 29 Photograph of a 4-$cm^2$ membrane–electrode-assembly (MEA) cell. ........ 36

Supplementary Fig. 30 Linear sweep voltammetry (LSV) curves of the $N_2$SN-functionalized Ag-Cu catalyst. The LSVs curves were measured in a 0.1 M $KHCO_3$ anolyte solution while supplying Ar (black) and $CO_2$ (red) to the cathode compartment of the MEA device. ........ 37

Supplementary Fig. 31 Electrocatalytic $CO_2$RR properties of the MEA electrolyzers using the different Ag-Cu catalysts. The corresponding Faradaic efficiency for the gas and liquid products on $N_2$SN-Ag-Cu (a), $N_3$N-Ag-Cu (b), $C_2$N-Ag-Cu (d), $C_3$-Ag-Cu (e) compared to pristine (c) catalysts at increasing cell voltages. The error bars represent the standard deviation of the measurements based on three independent samples. ........ 38

Supplementary Fig. 32 $CO_2$ electroreduction performance in the MEA electrolyzers. Faradaic efficiency for the $C_{2+}$ ($C_2H_4$, $C_2H_5OH$, n-propanol and acetate) (a), *j*–V plots of the partial current densities for the $C_1$ (CO, $CH_4$ and HCOOH) (b) and $H_2$ products (c) on $N_2$SN-Ag-Cu(pink), $N_3$N-Ag-Cu(blue), $C_2$N-Ag-Cu(green), $C_3$-Ag-Cu(orange) compared to pristine(gray) measured with a 0.1 M $KHCO_3$ anolyte solution. The error bars represent the standard deviation of three independent measurements. ........ 39

Supplementary Fig. 33 Enhancement factor of $FE_{C2+}$ for $N_2$SN-Ag-Cu (pink) and $N_3$N-Ag-Cu (blue) compared to pristine. ........ 40

Supplementary Fig. 34 Selectivity for $C_{1\text{-}2+}$ hydrocarbons on the different electrodes in the MEA electrolyzers. The estimated ratio for $\boldsymbol{jC2+}$ and $\boldsymbol{jC1}$ on the different Ag-Cu electrodes measured with a 0.1 M $KHCO_3$ anolyte solution. ........ 41

Supplementary Fig. 35 The influence of $CO_2$ gas flow rate on different products

selectivity of $N_2SN$-Ag-Cu at different voltages. (a) $C_2H_4$, (b) CO and (c) $H_2$. ...........42

Supplementary Fig. 36 SEM pictures of the $N_2SN$-Ag-Cu catalyst after electrolysis in MEA. ..........43

Supplementary Fig. 37 GC traces of the Calibration curve. The concentrations of $H_2$, CO, $CH_4$, $C_2H_4$, $C_2H_6$, $C_3H_6$, $C_3H_8$, and $C_4H_{10}$ is 0.25%, respectively, while the concentration of $CO_2$ is 98.0%. ..........44

Supplementary Fig. 38 Representative GC traces of the gas products formed on $N_2SN$-functionalized Ag-Cu. ..........45

Supplementary Table 1. Summary of the total energy and adsorption energy of the different configurations of the thiadiazole-functionalized Cu catalyst. The different configurations are presented in Supplementary Figure 12. ..........46

Supplementary Table 2. Summary of the calculated electrochemically active surface area (ECSA) for 15at.% Ag-Cu and $N_2SN$-15at.% Ag-Cu catalysts. ..........46

Supplementary Table 3. Summary of the XPS data for $N_2SN$-functionalized Ag-Cu at different reaction times. The atomic ratio N/Cu refers to the functionalization degree per Cu atom. ..........47

Supplementary Table 4. Summary of the Cu binding energy from the Auger $L_3M_{45}M_{45}$ transition modes for pristine, $N_2SN$-, $N_3N$-, $C_2N$-, $C_3$-functionalized Ag-Cu samples and $H_2O_2$-oxidized Ag-Cu. ..........47

Supplementary Table 5. Summary of the EXAFS data. $E_0$ and corresponding oxidation states (δ) of Cu. ..........47

Supplementary Table 6. EXAFS fitting parameters at the Cu K-edge for different samples ($S_0^2$=0.831). ..........48

Supplementary Table 7. Estimated ratios (in peak area) between the atop CO and the bridge CO obtained from the deconvoluted spectra[1,2]. The data represents the average values obtained from two independent sets of samples. ..........49

Supplementary Table 8. Summary of the estimated FEs for different groups functionalized Ag-Cu electrodes measured at different applied potentials in the H-cell reactors. The standard deviation of the measurements was estimated from three independent samples. ..........49

Supplementary Table 9. Summary of the FEs obtained on the different functionalized

Ag-Cu electrodes measured at different applied potentials in the MEA electrolyzers. The standard deviation of the measurements was estimated from three independent samples. ........ 51

**Supplementary Figures**

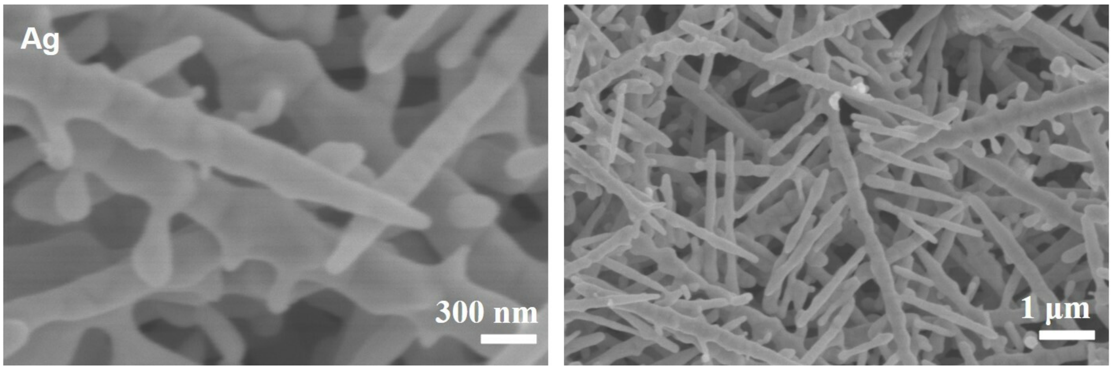


**Supplementary Fig.1 SEM images of pure Ag.**

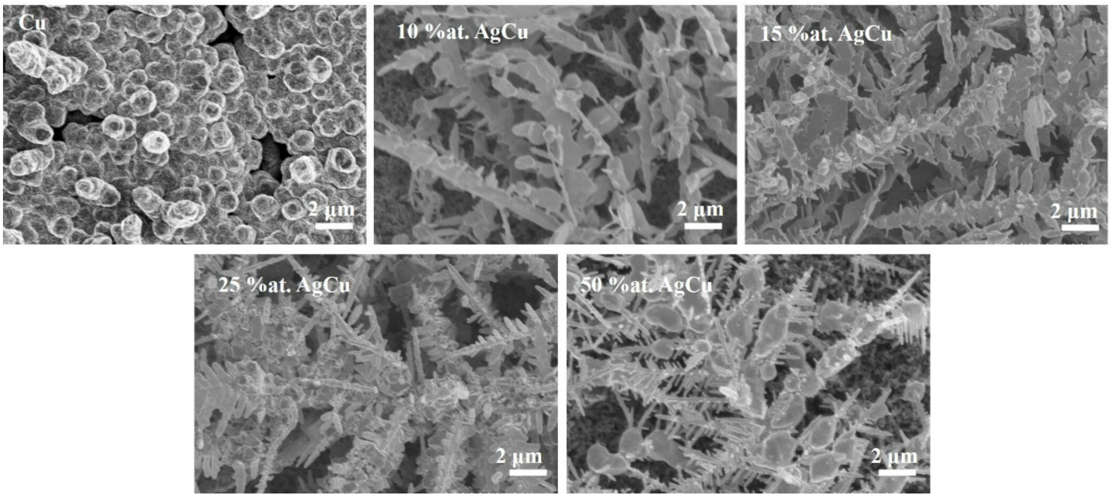


**Supplementary Fig. 2 SEM images of the Ag-Cu electrodes with different Ag atomic ratios.** SEM images of different atomic ratios of Ag in Ag-Cu electrodes（0%at., 10 %at., 15 %at., 25 %at. and 50 %at.）.

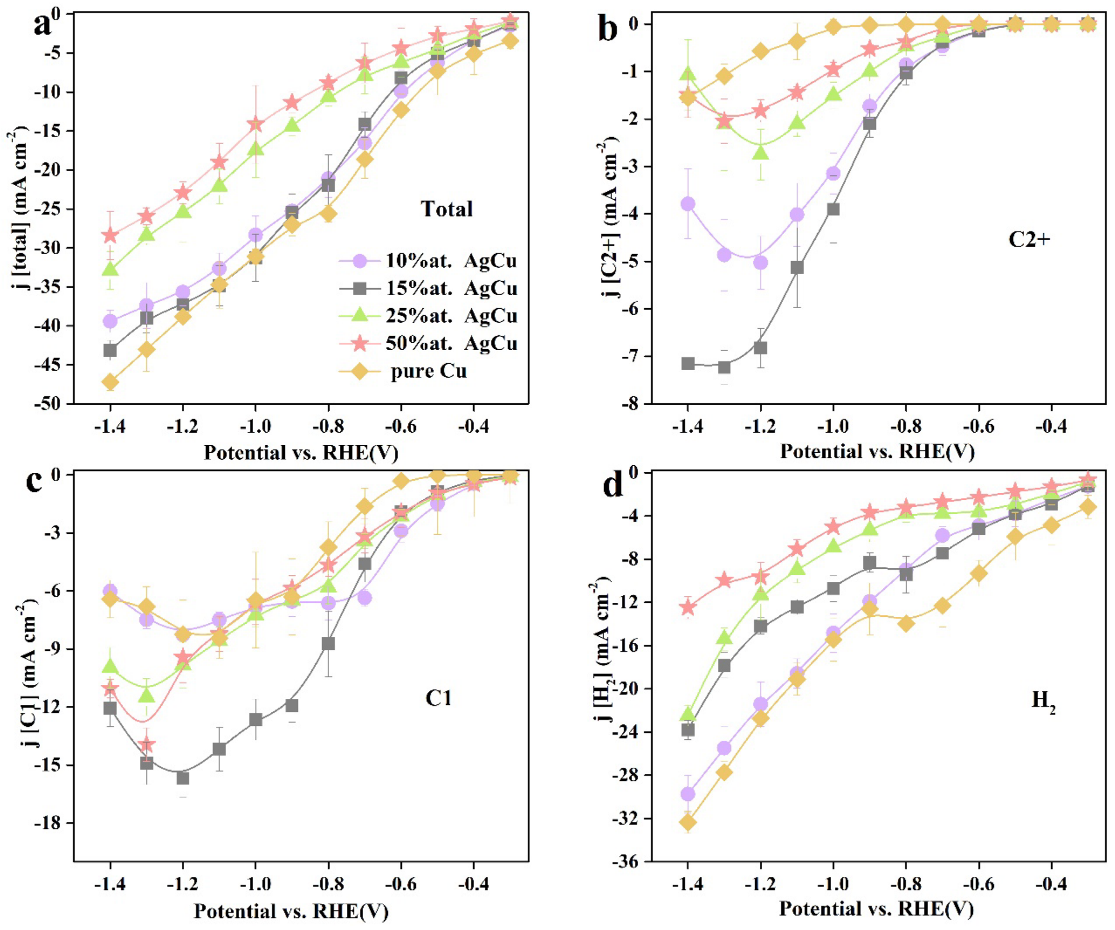


**Supplementary Fig. 3 Comparisons of the current density on the different catalysts measured in the H-cell reactors.** The current density for the different Ag atomic ratios in Ag-Cu catalysts (0%at. AgCu (yellow), 10%at. AgCu (purple), 15%at. AgCu (gray), 25%at. AgCu (green), and 50%at. AgCu(red)). The error bars represent the standard deviation of three independent samples during the entire operation and for each cell voltage.

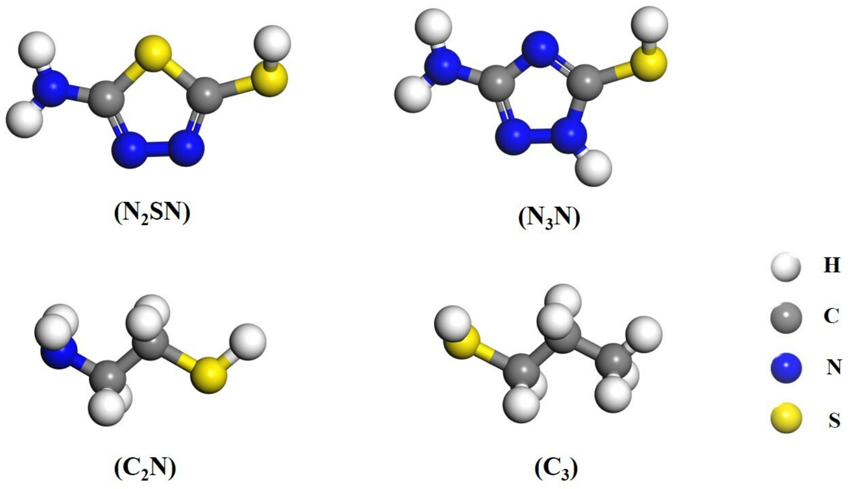


**Supplementary Fig. 4 Molecular structures of the different molecules used for the functionalization of Ag-Cu.**

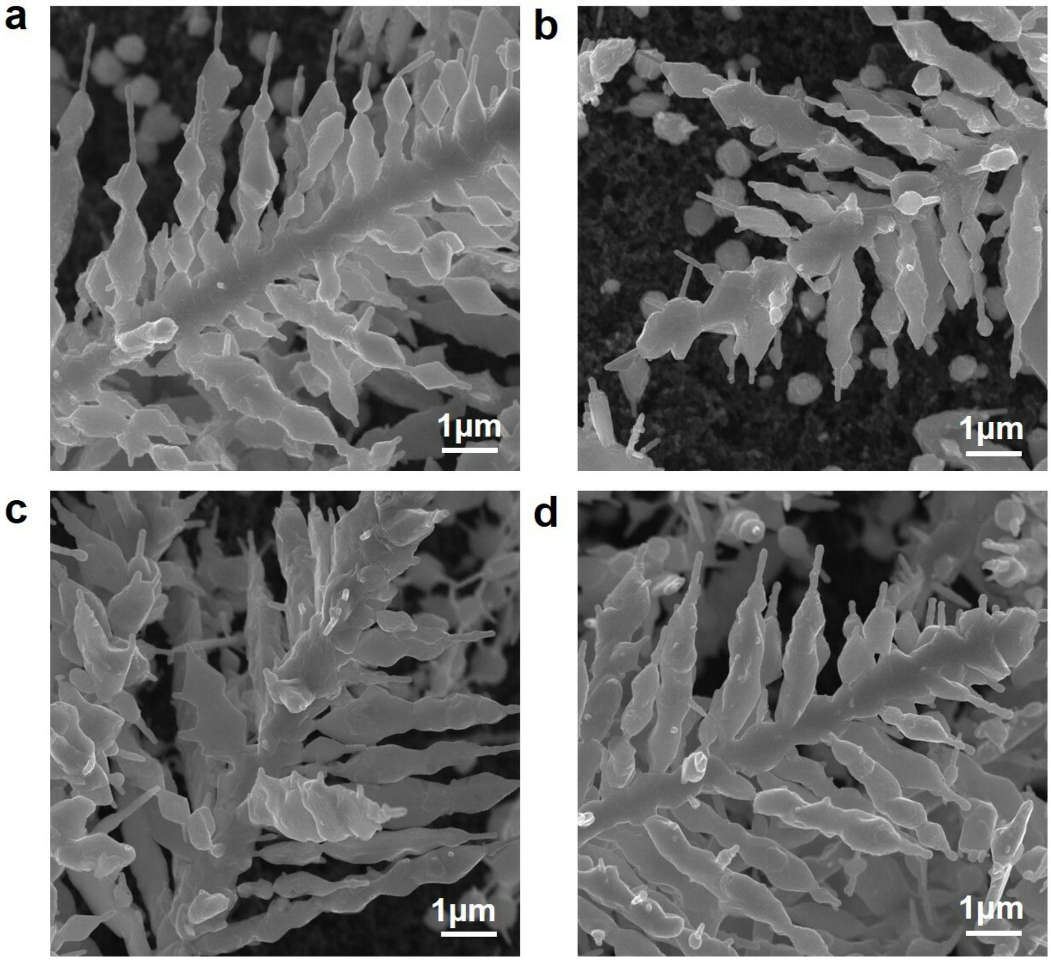


**Supplementary Fig. 5 SEM figures for the pristine and functionalized Ag-Cu.** SEM figures of different functional groups modified 15 %at. Ag-Cu electrodes: P (a), (b) $N_3N$, (c) $C_2N$ and (d) $C_3$.

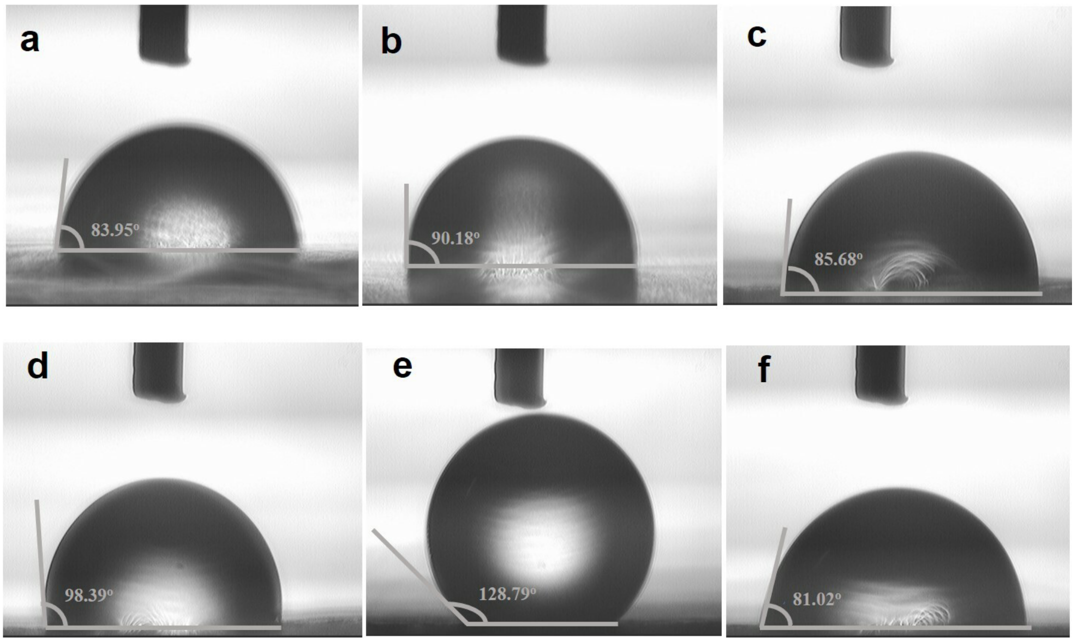


**Supplementary Fig. 6 Wettability of the pristine and functionalized Ag-Cu electrodes.** The water contact angles measured for 15 %at. Ag-Cu: P (a), $N_2SN$ (b), $N_3N$ (c), $C_2N$ (d), $C_3$ (e) and $N_2SS$ (f) before $CO_2RR$.

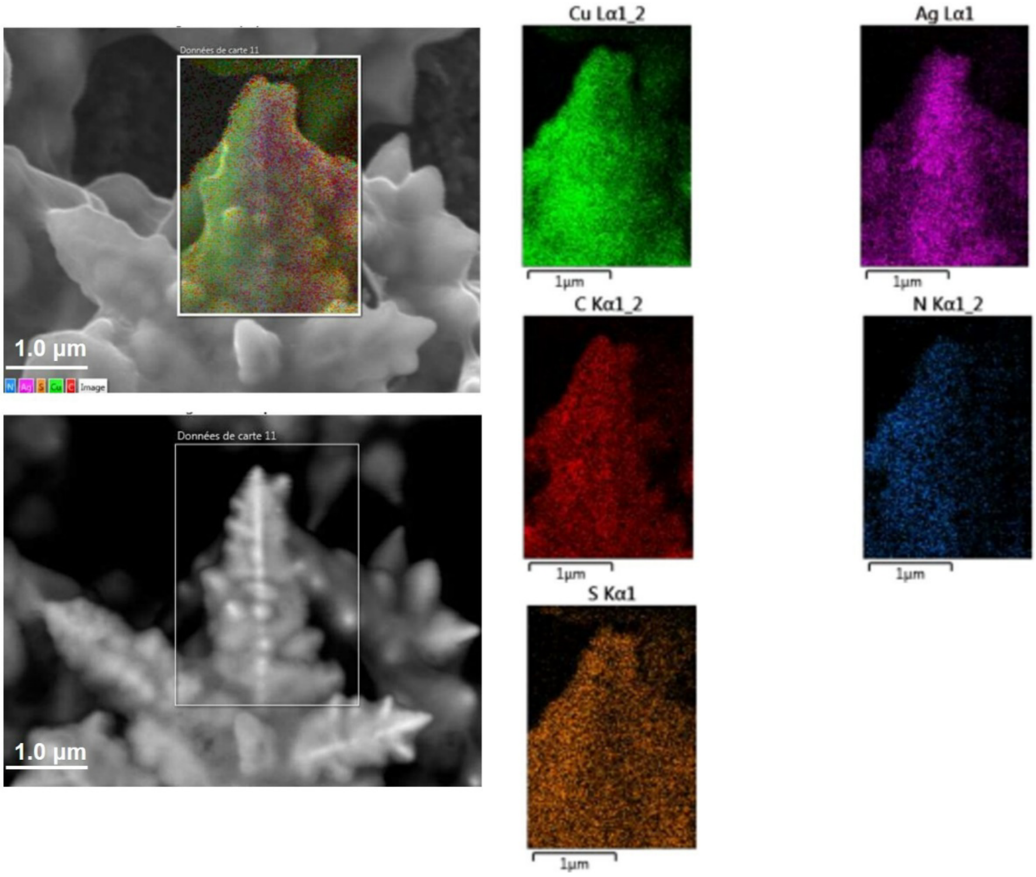


**Supplementary Fig. 7 Structural and compositional analyses of the 15 %at. Ag-Cu-$N_2$SN catalyst.** Low magnification SEM images (top left panel) and the related EDX elemental mapping of Cu (green), Ag (purple), C (red), N (dark blue), and S(orange) (right panels).

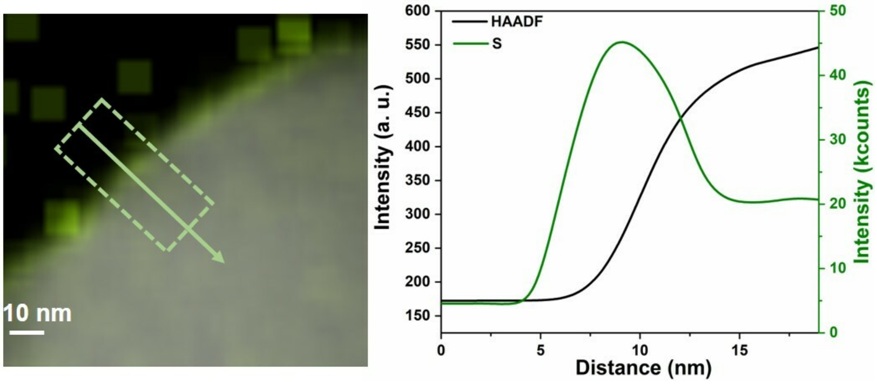


**Supplementary Fig. 8 HAADF-STEM data of $N_2$SN-functionalized Ag-Cu catalyst.** *Right panel:* The superposition of the HAADF-STEM image of the $N_2$SN functionalized Ag-Cu ultrathin section with the sulfur (S) EDS elemental map. The arrow highlights the area used to extract the intensity profiles. *Left panel:* The corresponding intensity profiles of the HAADF-STEM images and S elemental map.

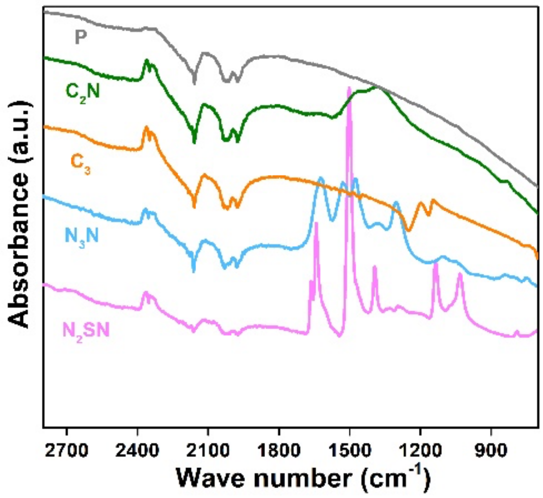


**Supplementary Fig. 9 Fourier transformed infrared (FTIR) spectra of the pristine functional groups.** The ATR-FTIR spectra of pristine (non-functionalized) 15 %at. Ag-Cu (gray), 15 %at. Ag-Cu-$N_2SN$ (purple), 15 %at. Ag-Cu-$N_3N$ (blue), 15 % at. Ag-Cu-$C_2N$ (green) and 15 %at. Ag-Cu-$C_3$ (orange) before $CO_2RR$.

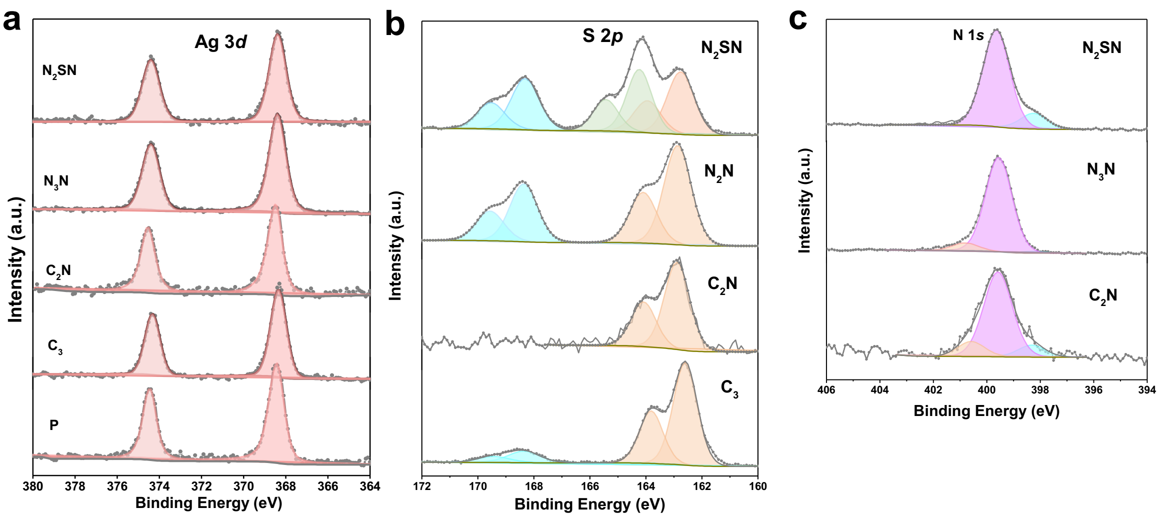


**Supplementary Fig. 10 X-ray photoelectron spectra (XPS) spectra of the different Ag-Cu catalysts.** The high-resolution XPS spectra of the Ag3*d* (a), S2*p* (b) and N1*s* (c) regions of the different functionalized 15 %at. Ag-Cu catalysts before reaction**.**

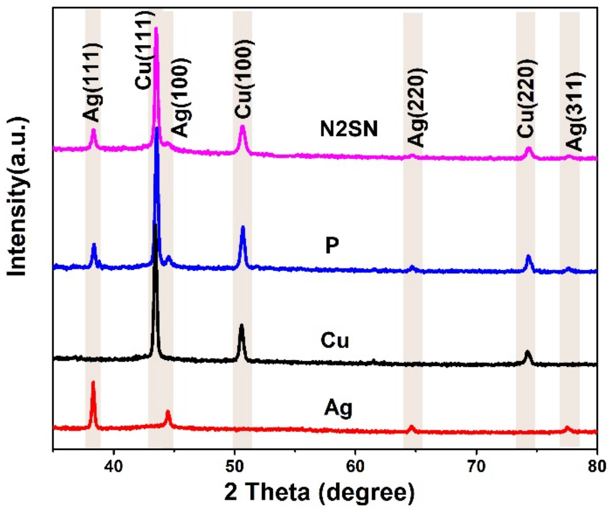


**Supplementary Fig. 11 X-ray diffraction (XRD) data of the different Ag-Cu catalysts.** Powder XRD spectra of 15 %at. Ag-Cu and 15 %at. Ag-Cu-$N_2$SN compared with Cu, Ag metals used as references.

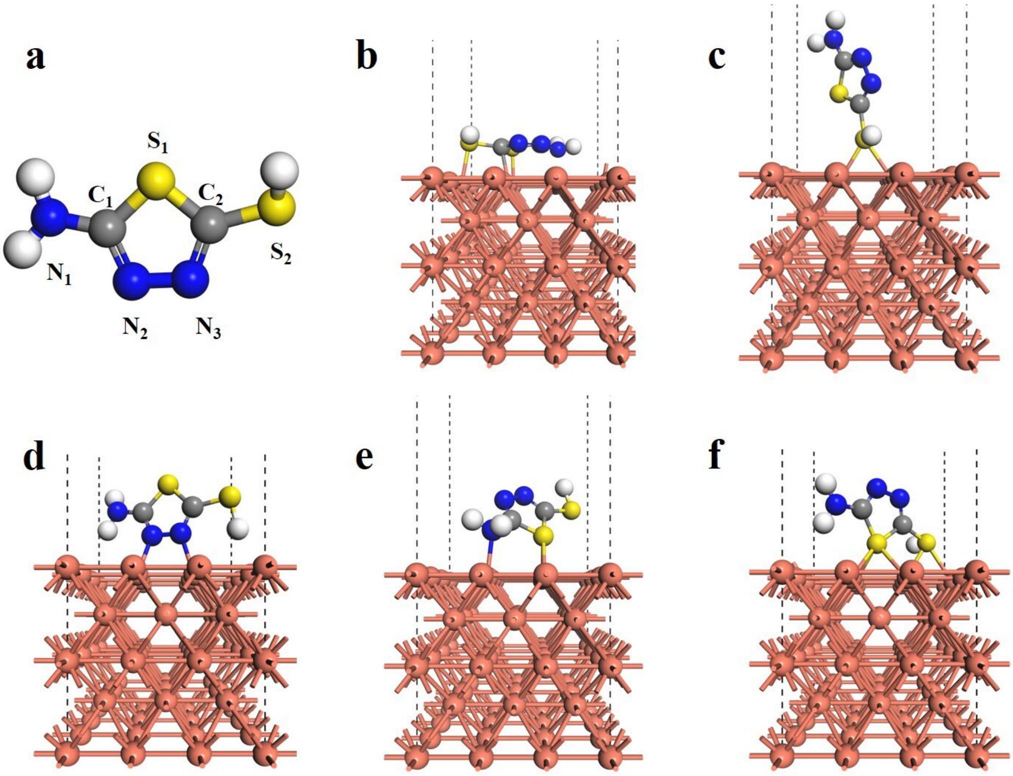


**Supplementary Fig. 12 Schematic representations of the different configurations of $N_2$SN on Cu (side view).** Molecular structure of $N_2$SN (a), $S_1$-$C_2$-$S_2$ flat model (b), $S_1$ model (c), $N_2$-$N_3$ model (d), $N_1$-$S_1$ model (e) and $S_1$-$S_2$ model (f).

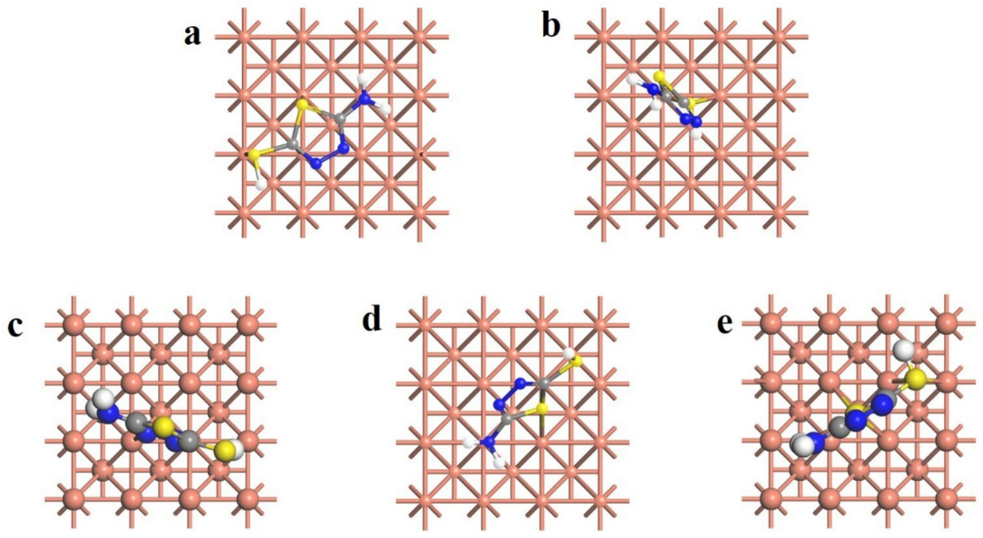


**Supplementary Fig. 13 The different configurations of $N_2SN$ on Cu (top view) used for the calculation results summarized in Supplementary Table 1.** $S_1$-$C_2$-$S_2$ flat model (a), $S_1$ model (b), $N_2$-$N_3$ model (c), $N_1$-$S_1$ model (d) and $S_1$-$S_2$ model (e).

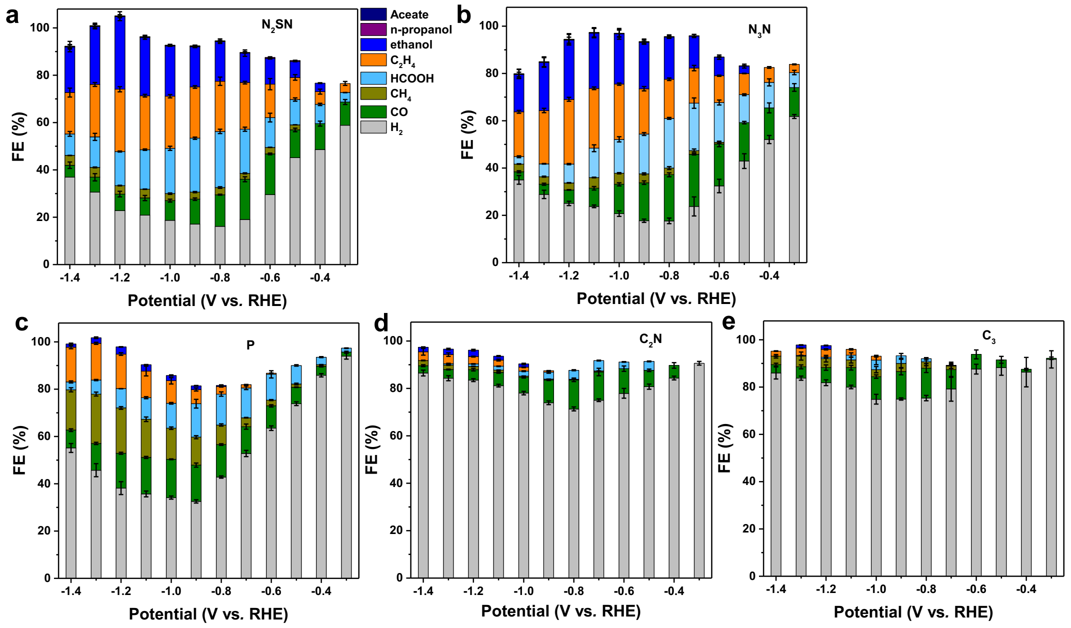


**Supplementary Fig. 14 Comparisons of the Faradaic efficiencies on the different catalysts measured in the H-cell reactors.** The Faradaic efficiency for the different products on N2SN-(a), N3N-(b), C2N-(d) and C3-(e) Ag-Cu electrodes, as well as pristine sample (c). The error bars represent the standard deviation of three independent samples during the entire operation and for each cell voltage.

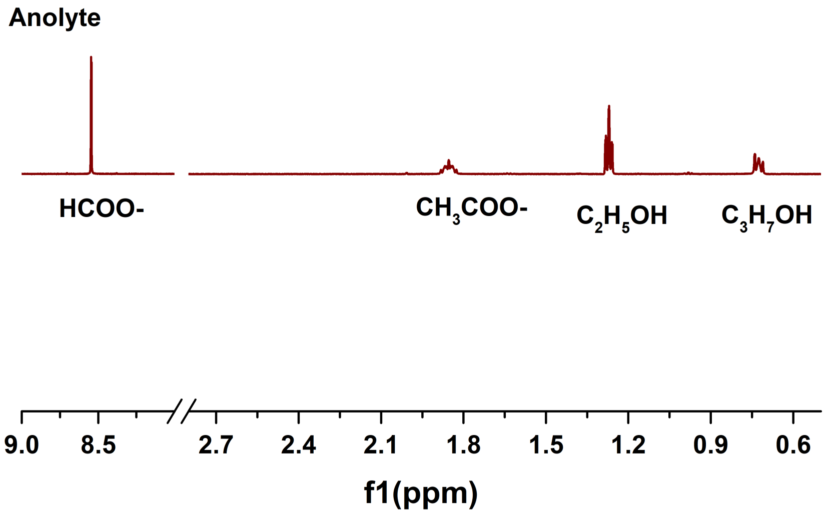


**Supplementary Fig.15 Representative NMR spectra of the liquid products formed on $N_2SN$-functionalized Ag-Cu.** $^1$H NMR spectra of liquid products collected from the electrolyte after 30 min at –1.2 V versus the reversible hydrogen electrode (vs. RHE).

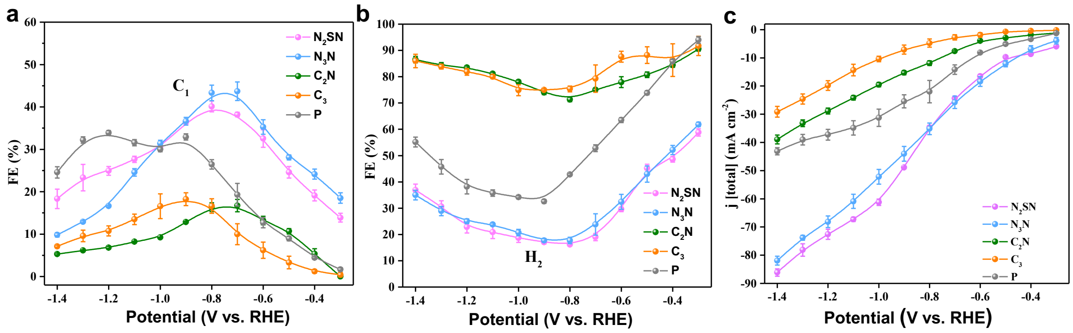


**Supplementary Fig. 16 $CO_2$RR performance in the H-cell reactors. a, b** FE values for $C_1$ products (**a**) and $H_2$ (**b**) on the different catalysts at various potentials ranging from -0.3 to −1.4 V vs. RHE in 0.5 M $KHCO_3$. c, *j*–V plots of the total current densities versus the RHE on different samples in 0.5 M $KHCO_3$. The error bars represent the standard deviation of three independent measurements.

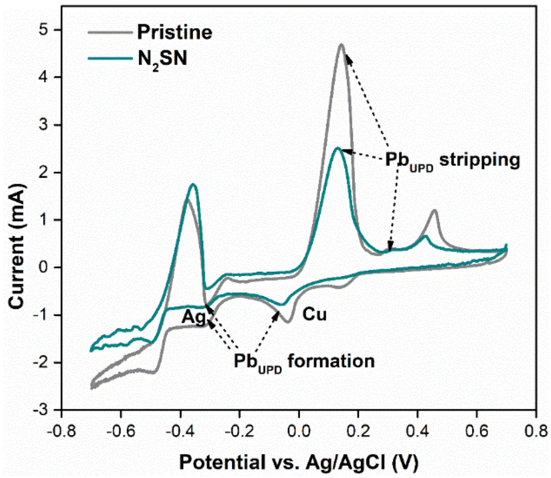


**Supplementary Fig. 17 CVs for different samples measured in 100 mM $HClO_4$ + 1 mM $Pb(ClO_4)_2$.**

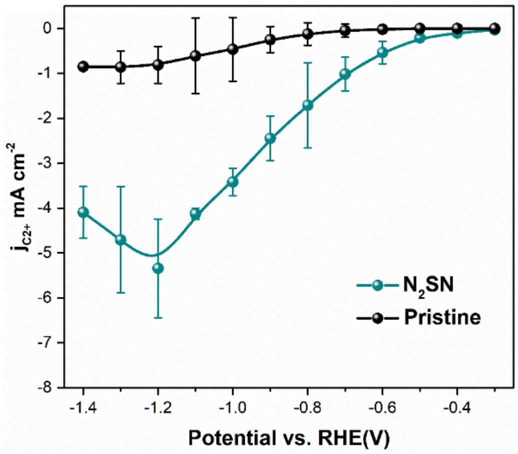


**Supplementary Fig. 18 Partial $C_{2+}$ products current density normalized to Cu ECSA for 15at.% Ag-Cu(gray) and $N_2$SN-15at.% Ag-Cu (dark blue) catalysts versus potential for $CO_2$RR in H-cell.** The error bars represent the standard deviation of three independent measurements.

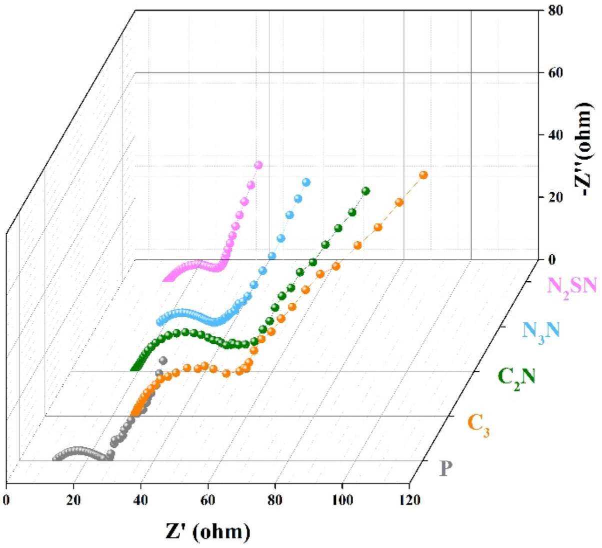


**Supplementary Fig. 19 Electrochemical impedance spectroscopy (EIS) measured for the different Ag-Cu catalysts.** The EIS spectra measured in the H-cell configuration for pristine (non-functionalized) 15 %at. Ag-Cu (gray), 15 %at. Ag-Cu-N2SN (purple), 15 %at. Ag-Cu-N3N (blue), 15 % at. Ag-Cu-C2N (green) and 15 % at. Ag-Cu-C3 (orange). The EIS data were recorded in $CO_2$-saturated 0.5 M $KHCO_3$ solution.

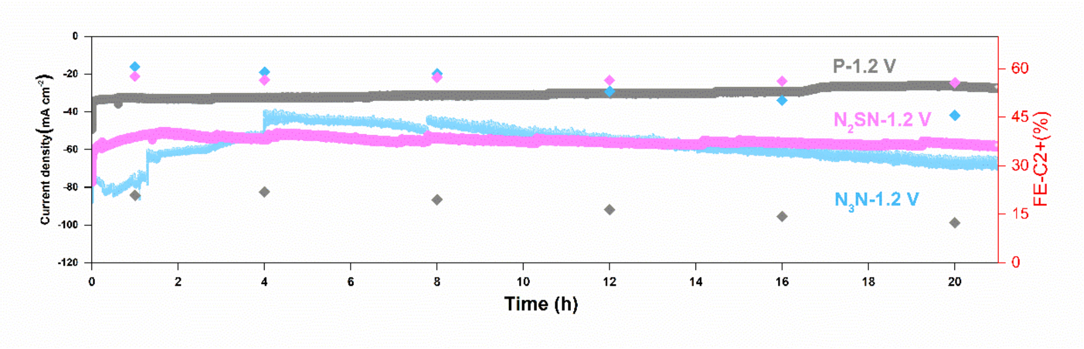


**Supplementary Fig. 20 Stability measurements of N$_2$SN-, N$_3$N- functionalized Ag-Cu compared with pristine Ag-Cu measured in the H-cell reactors.** The stability of Ag-Cu-N$_2$SN (pink), Ag-Cu-N$_3$N (blue) and Ag-Cu (gray) were obtained at -1.2 V vs. RHE without *iR*-correction.

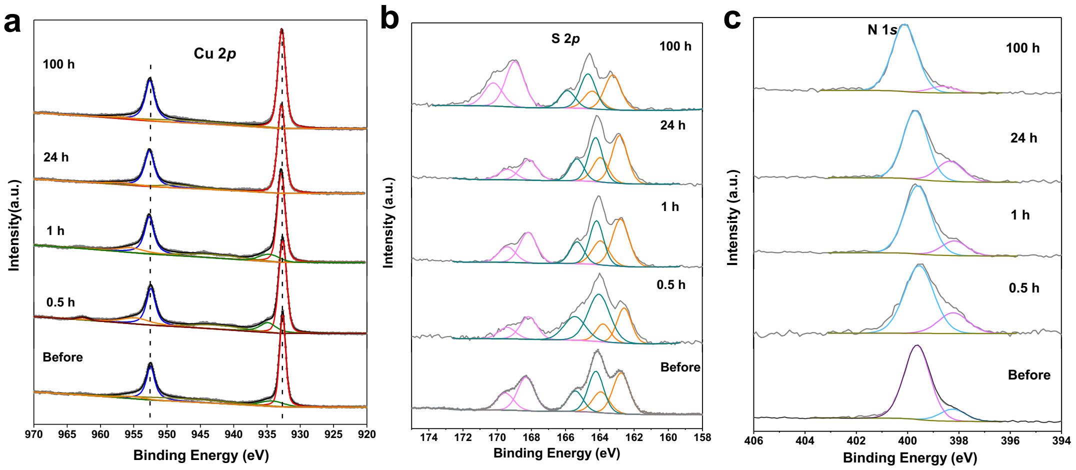


**Supplementary Fig. 21 X-ray photoelectron spectra of $N_2SN$ before and after $CO_2RR$.** The XPS data from the Cu 2*p* (a), S 2*p* (b) and N1*s* (c) regions were measured before and after operation up to 100 h at -1.2 V vs. RHE.

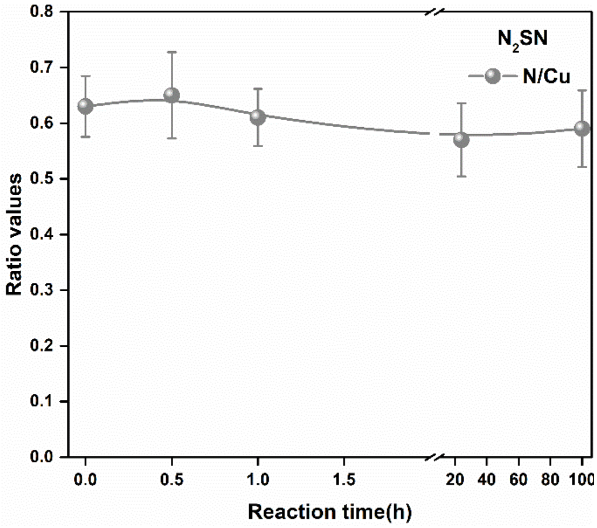


**Supplementary Fig. 22 Estimated atomic N/Cu ratio of the $N_2$SN-Ag-Cu electrodes along the $CO_2$RR operating time.** The atomic N/Cu ratio of $N_2$SN-Ag-Cu were estimated from the deconvoluted XPS spectra shown in Supplementary Figure 21. The error bars represent the standard deviation of three independent measurements.

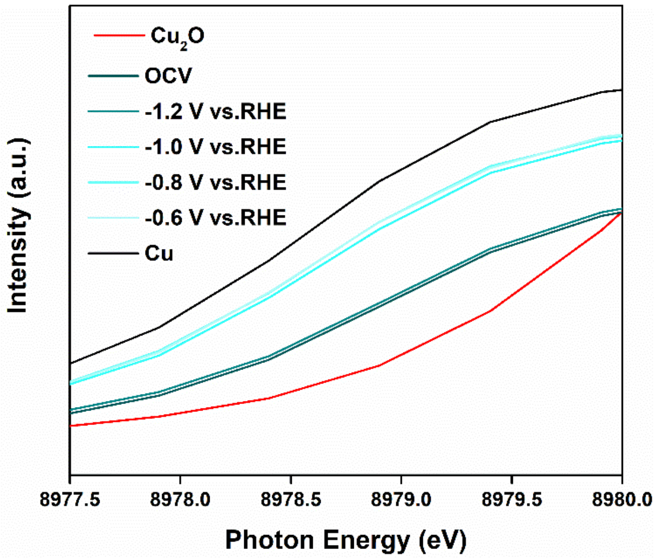


**Supplementary Fig. 23 Cu K-edge X-ray absorption near edge structure (XANES) spectra of the different Ag-Cu catalysts.** Magnification of the *operando* Cu K-edge XANES spectra of $N_2SN$-functionalized Ag-Cu electrode during $CO_2RR$. The operando XANES measurements were performed after applying a fixed potential vs. RHE for 30 minutes.

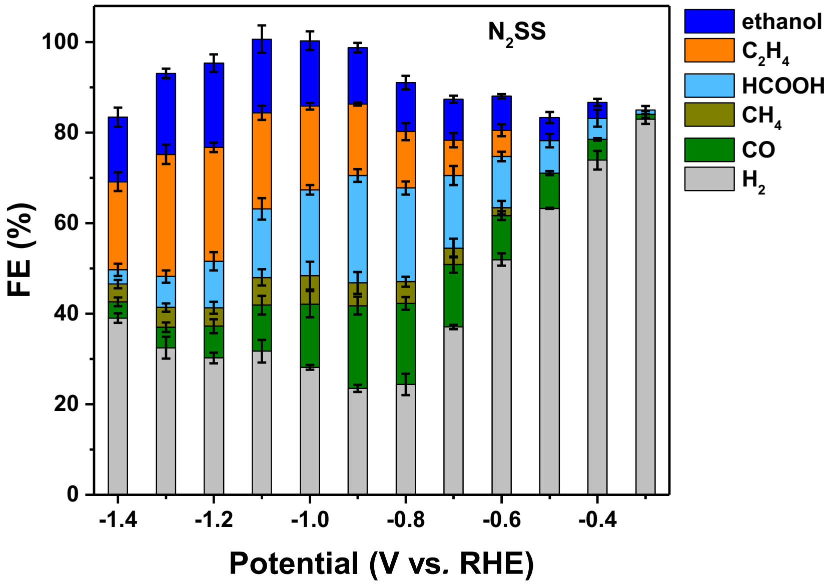


**Supplementary Fig. 24 The Faradaic efficiency for the different products on $N_2$SS functionalized 15at.% Ag-Cu catalyst.** The error bars represent the standard deviation of three independent measurements.

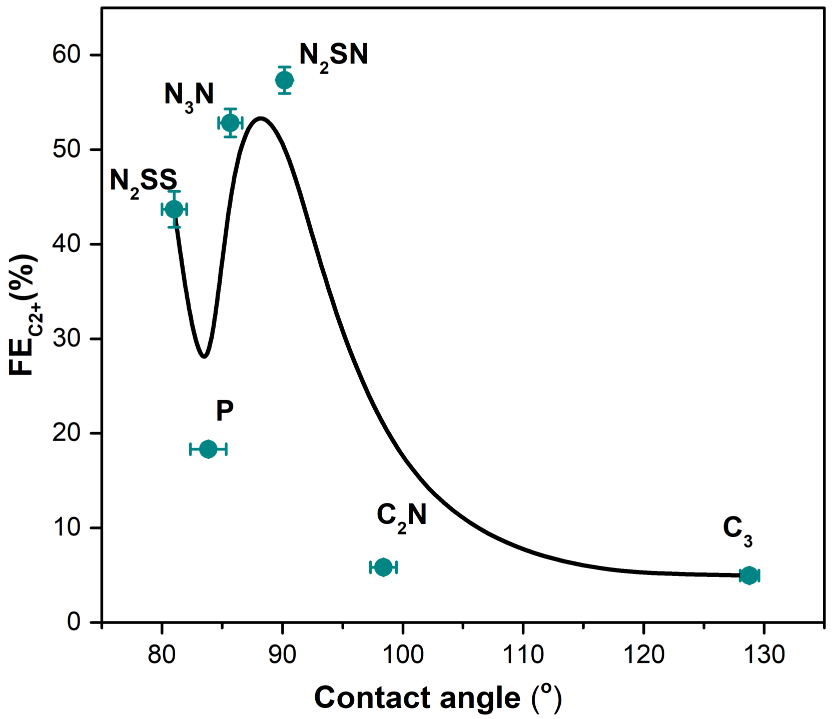


**Supplementary Fig. 25 The relationship between water contact angle and Faradaic efficiency of $C_{2+}$ products in H-cell for 15at.% Ag-Cu(P), $N_2SN$, $N_3N$, $C_2N$, $C_3$ and $N_2SS$.** The error bars represent the standard deviation of three independent measurements.

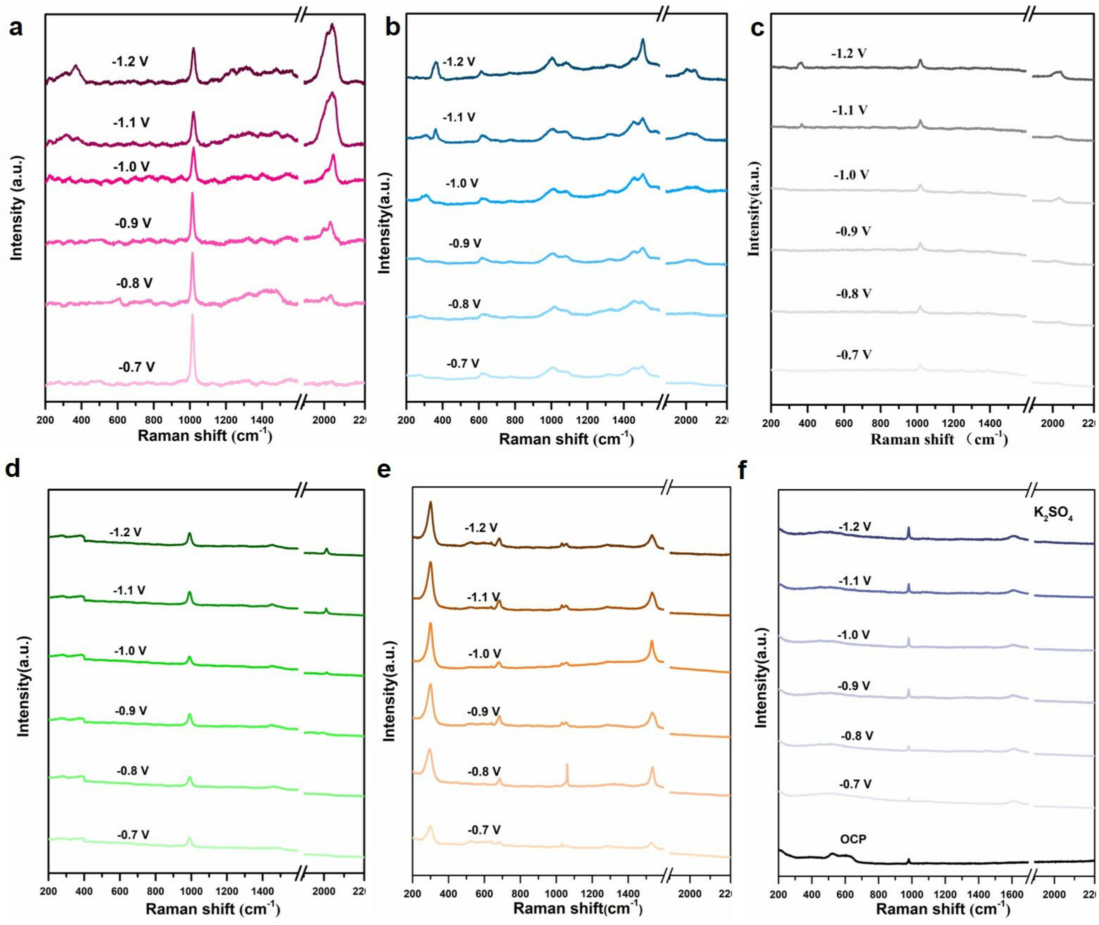


**Supplementary Fig. 26 Operando Raman spectra of (a) $N_2$SN-, (b) $N_3$N-, (c) $C_2$N-, and (d) $C_3$-functionalized Ag-Cu electrodes compared with (e) pristine Ag-Cu. a-e**, The operando Raman measurements were carried out between -0.7 V and -1.2 V vs. RHE in a $CO_2$-saturated $KHCO_3$ solution. To confirm that the signals are solely coming from the $CO_2$RR, the $N_2$SN-functionalized Ag-Cu catalyst was also tested in an Ar-saturated $K_2SO_4$ electrolyte solution (**f**).

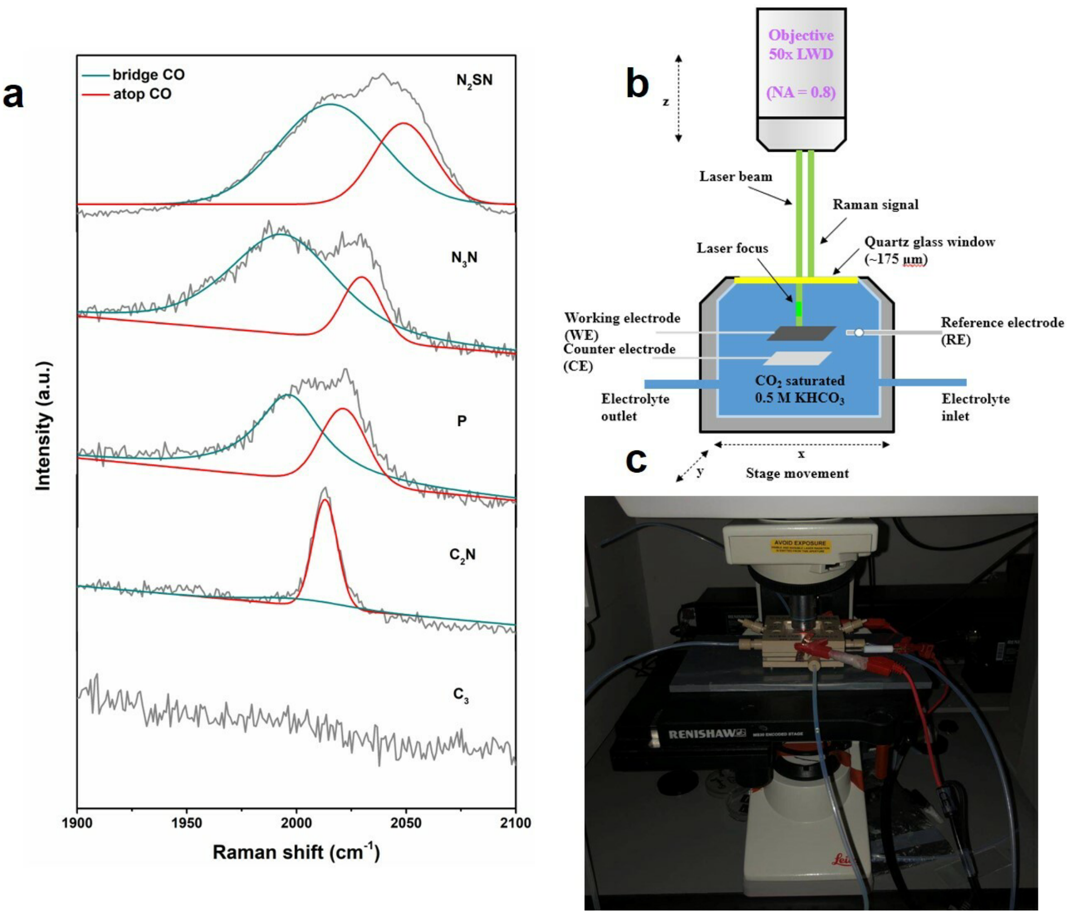


**Supplementary Fig. 27 Deconvolution of the Raman signals around 2000 cm$^{-1}$.** *Right panel:* Operando Raman spectra centered around 2000 cm$^{-1}$ representing the C≡O stretch region on pristine and functionalized Ag-Cu electrodes. The asymmetric signals were deconvoluted into two components for the atop and the bridge CO using Lorentzian curves. The ratio of the intensities (in peak area) of the two bands is summarized in Supplementary Table 7. *Left panel:* Schematic representation and photograph of the operando setup.

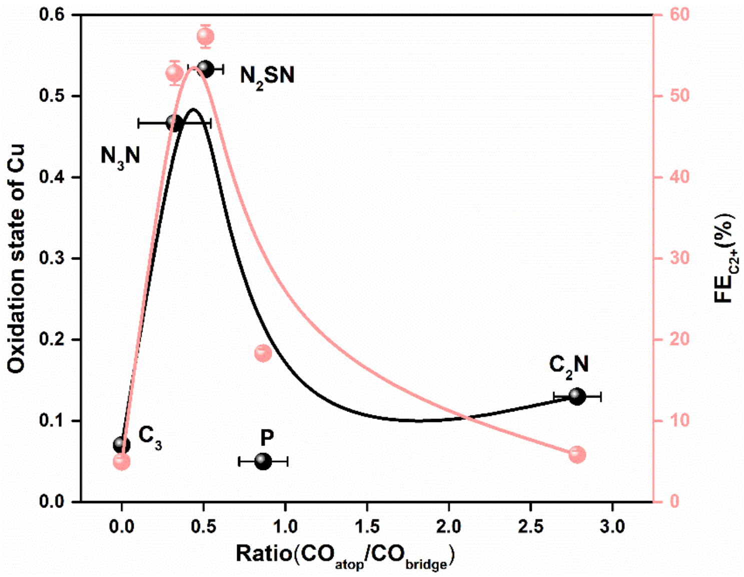


**Supplementary Fig. 28 Relationship between the ratio of $CO_{atop}$ and $CO_{bridge}$ with the oxidation degree of Cu obtained from our XANES measurements.** The error bars represent the standard deviation of three independent measurements.

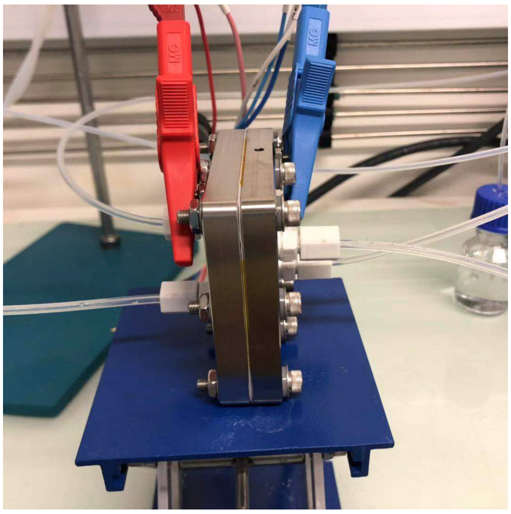

**Supplementary Fig. 29 Photograph of a 4-cm$^2$ membrane–electrode-assembly (MEA) cell.**

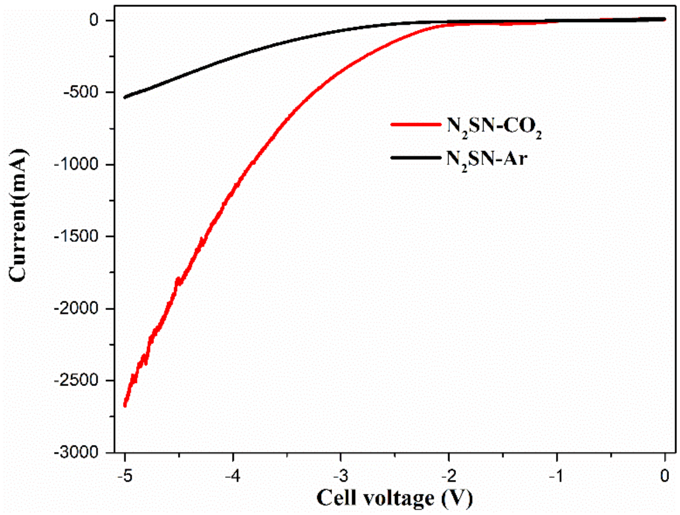


**Supplementary Fig. 30 Linear sweep voltammetry (LSV) curves of the $N_2$SN-functionalized Ag-Cu catalyst.** The LSVs curves were measured in a 0.1 M $KHCO_3$ anolyte solution while supplying Ar (black) and $CO_2$ (red) to the cathode compartment of the MEA device.

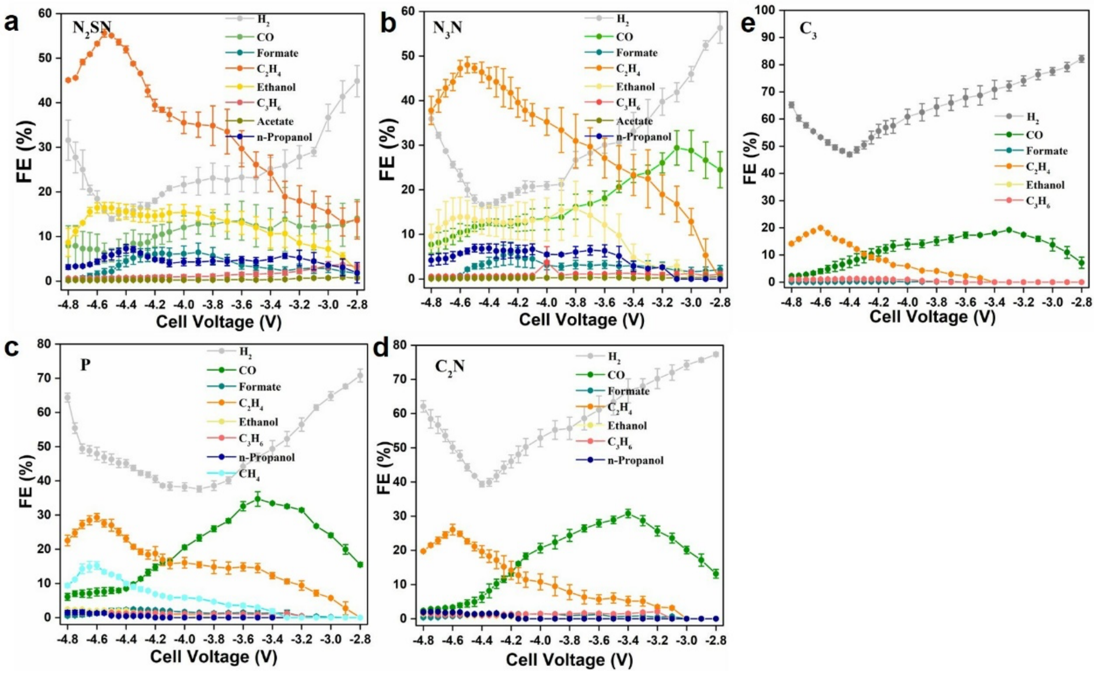


**Supplementary Fig. 31 Electrocatalytic $CO_2$RR properties of the MEA electrolyzers using the different Ag-Cu catalysts.** The corresponding Faradaic efficiency for the gas and liquid products on $N_2$SN-Ag-Cu (a), $N_3$N-Ag-Cu (b), $C_2$N-Ag-Cu (d), $C_3$-Ag-Cu (e) compared to pristine (c) catalysts at increasing cell voltages. The error bars represent the standard deviation of the measurements based on three independent samples.

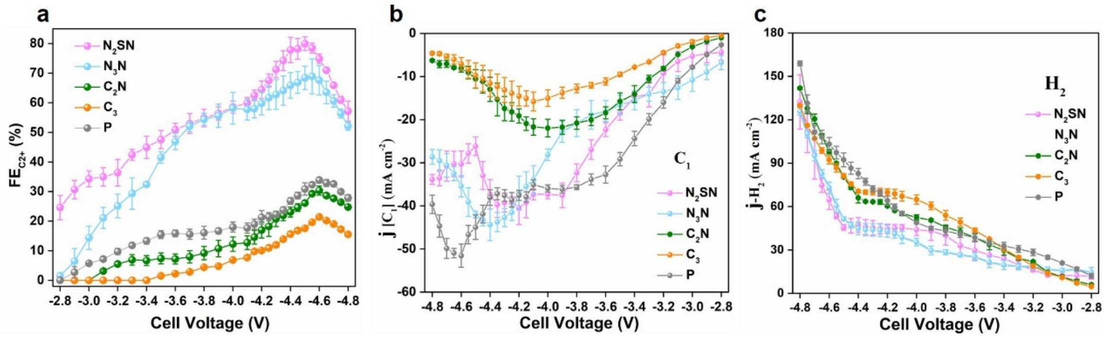


**Supplementary Fig. 32 $CO_2$ electroreduction performance in the MEA electrolyzers.** Faradaic efficiency for the $C_{2+}$ ($C_2H_4$, $C_2H_5OH$, n-propanol and acetate) (a), *j*–V plots of the partial current densities for the $C_1$ (CO, $CH_4$ and HCOOH) (b) and $H_2$ products (c) on $N_2SN$-Ag-Cu(pink), $N_3N$-Ag-Cu(blue), $C_2N$-Ag-Cu(green), $C_3$-Ag-Cu(orange) compared to pristine(gray) measured with a 0.1 M $KHCO_3$ anolyte solution. The error bars represent the standard deviation of three independent measurements.

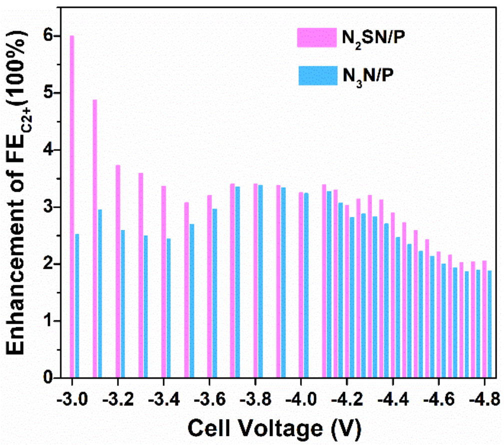


**Supplementary Fig. 33 Enhancement factor of $FE_{C2+}$ for $N_2SN$-Ag-Cu (pink) and $N_3N$-Ag-Cu (blue) compared to pristine.**

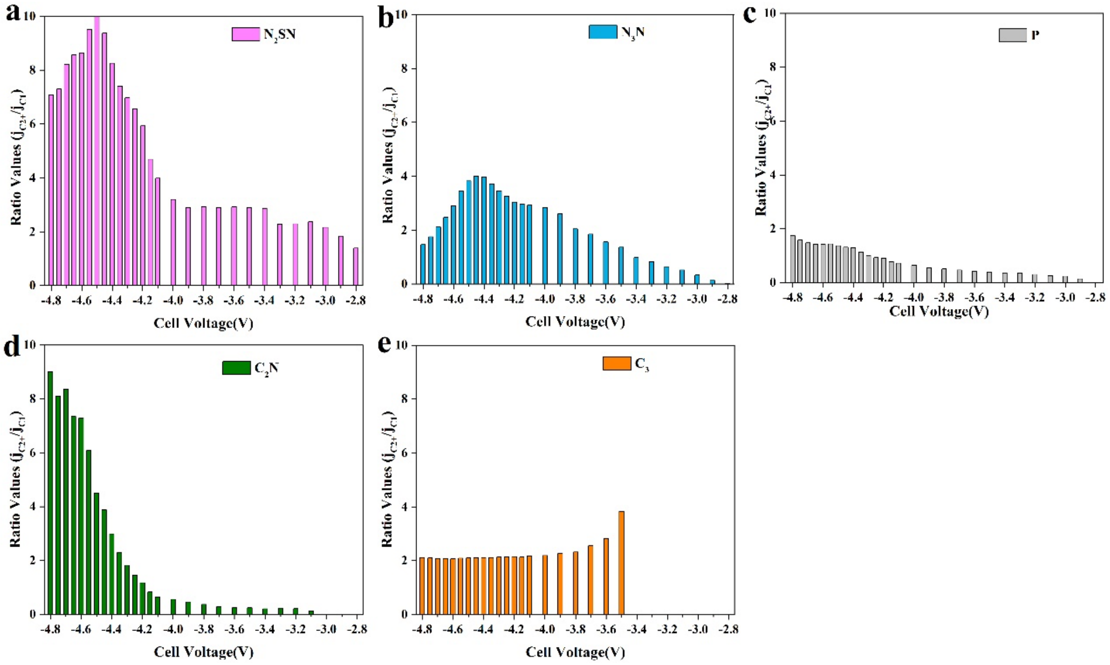


**Supplementary Fig. 34 Selectivity for $C_{1-2+}$ hydrocarbons on the different electrodes in the MEA electrolyzers.** The estimated ratio for $j_{C_{2+}}$ and $j_{C_1}$ on the different Ag-Cu electrodes measured with a 0.1 M $KHCO_3$ anolyte solution.

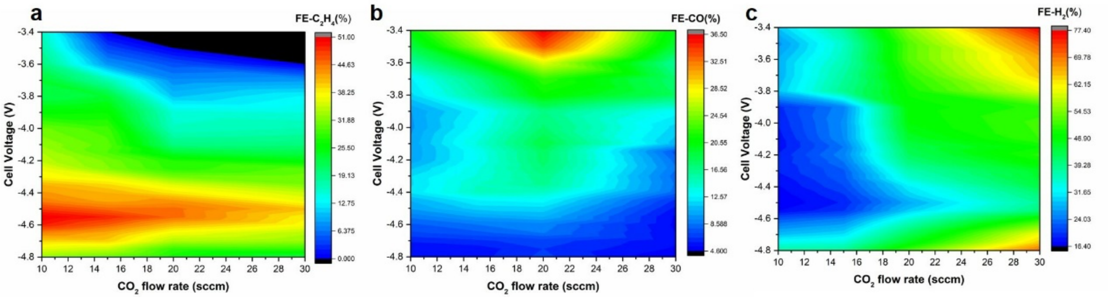


**Supplementary Fig. 35 The influence of $CO_2$ gas flow rate on different products selectivity of $N_2$SN-Ag-Cu at different voltages. (a) $C_2H_4$, (b) CO and (c) $H_2$.**

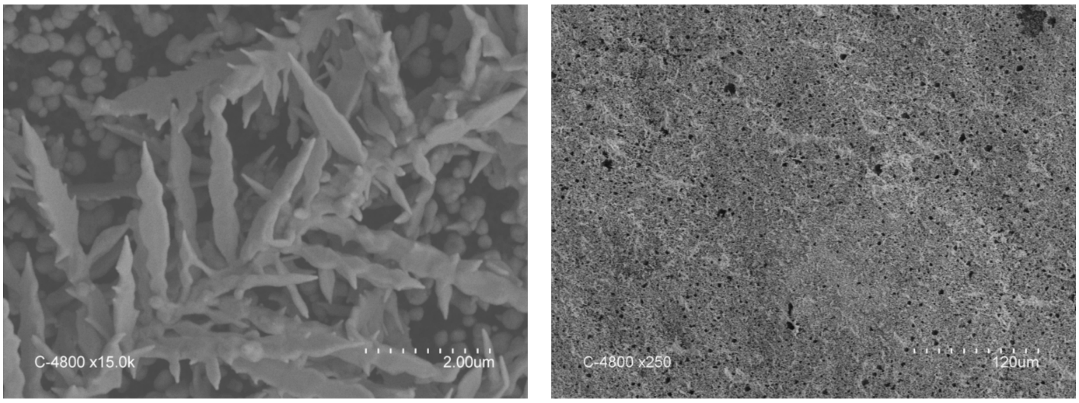


**Supplementary Fig. 36 SEM pictures of the $N_2$SN-Ag-Cu catalyst after electrolysis in MEA.**

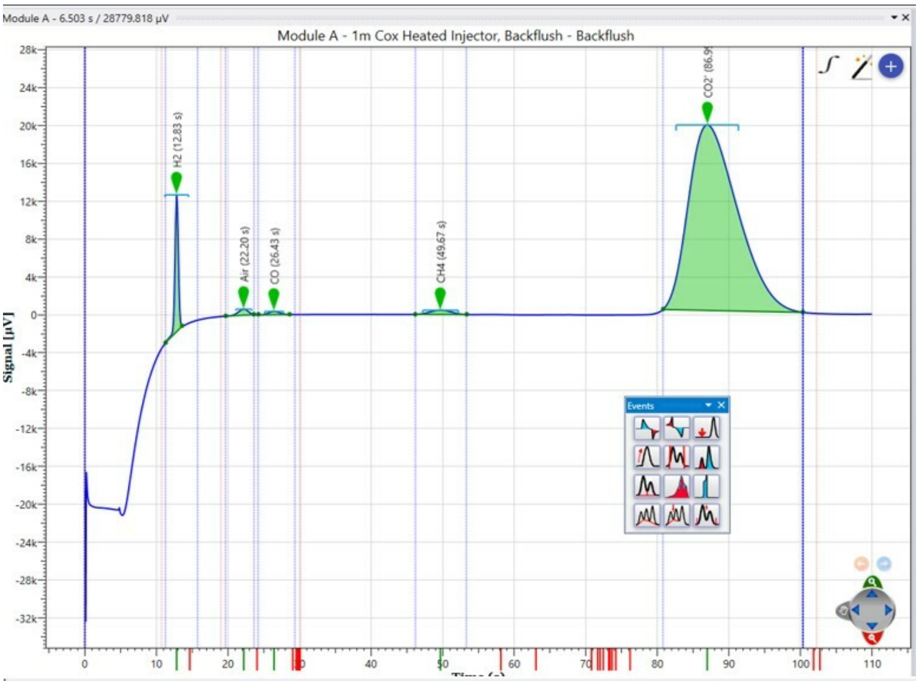


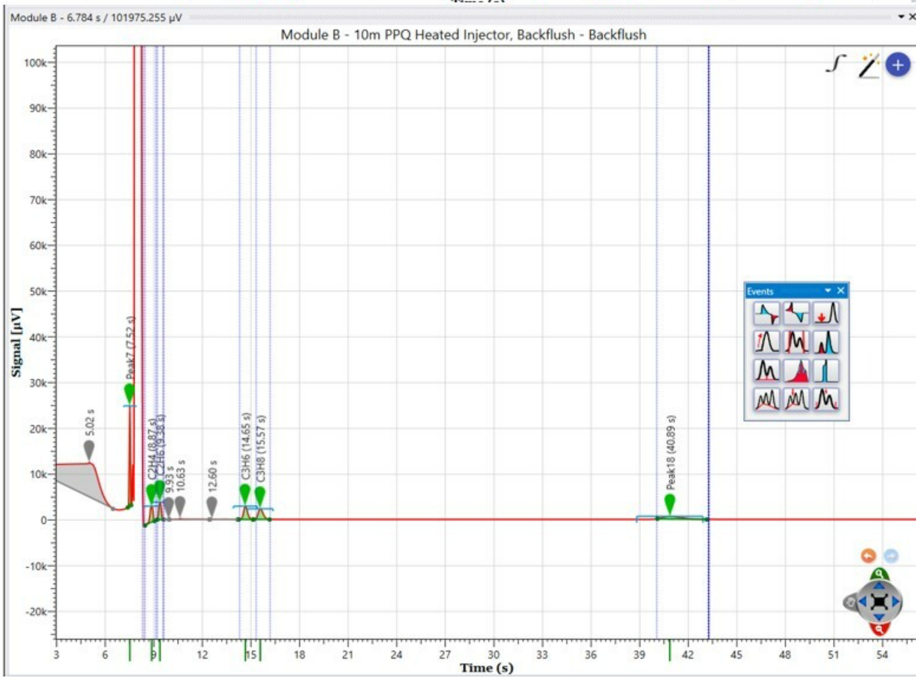


**Supplementary Fig. 37 GC traces of the Calibration curve.** **The concentrations of $H_2$, CO, $CH_4$, $C_2H_4$, $C_2H_6$, $C_3H_6$, $C_3H_8$, and $C_4H_{10}$ is 0.25%, respectively, while the concentration of $CO_2$ is 98.0%.**

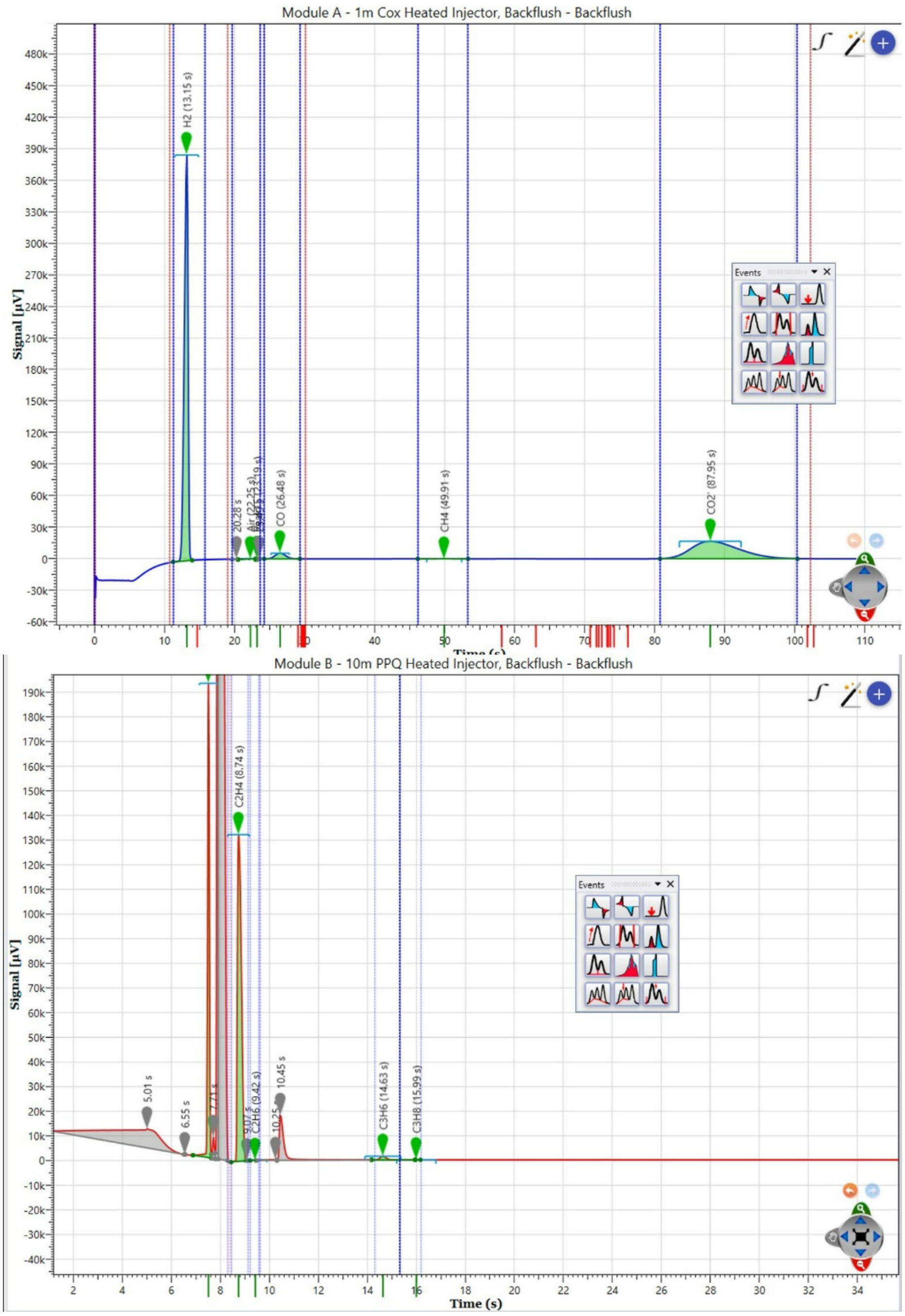


**Supplementary Fig. 38 Representative GC traces of the gas products formed on $N_2SN$-functionalized Ag-Cu.**

**Supplementary Table 1. Summary of the total energy and adsorption energy of the different configurations of the thiadiazole-functionalized Cu catalyst.** The different configurations are presented in Supplementary Figure 12.

| **Configuration** | **Energy (eV)** | **Slab (eV)** | **Functional molecular (eV)** | **Adsorption energy (eV)** |
|---|---|---|---|---|
| $S_1$-$C_2$-$S_2$ | -222.80 | -161.30 | -60.79 | -0.71 |
| $S_1$ | -222.40 | -161.30 | -60.79 | -0.30 |
| $N_2$-$N_3$ | -223.58 | -161.30 | -60.79 | -1.08 |
| $N_1$-$S_1$ | -222.53 | -161.30 | -60.79 | -0.44 |
| $S_1$-$S_2$ | -222.63 | -161.30 | -60.79 | -0.53 |

**Supplementary Table 2. Summary of the calculated electrochemically active surface area (ECSA) for 15at.% Ag-Cu and $N_2$SN-15at.% Ag-Cu catalysts.**

| **Sample** | **ECSA (Cu, $cm^2$)** | **ECSA (Ag, $cm^2$)** |
|---|---|---|
| 15at.% Ag-Cu | 8.4 | $5.1\times10^{-3}$ |
| $N_2$SN-15at.% Ag-Cu | 7.8 | $1.9\times10^{-3}$ |

**Supplementary Table 3. Summary of the XPS data for $N_2SN$-functionalized Ag-Cu at different reaction times.** The atomic ratio N/Cu refers to the functionalization degree per Cu atom.

| | Cu (at.%) | N (at.%) | Atomic Ratios of N/Cu |
|---|---|---|---|
| **Before** | 9.98 | 6.28 | 0.63 |
| **0.5 h** | 12.97 | 8.43 | 0.65 |
| **1 h** | 9.89 | 6.03 | 0.61 |
| **24 h** | 18.53 | 10.56 | 0.57 |
| **100 h** | 15.10 | 8.91 | 0.59 |

**Supplementary Table 4. Summary of the Cu binding energy from the Auger $L_3M_{45}M_{45}$ transition modes for pristine, $N_2SN$-, $N_3N$-, $C_2N$-, $C_3$-functionalized Ag-Cu samples and $H_2O_2$-oxidized Ag-Cu.**

| Samples | $^1G$ (eV) | $^3F$ (eV) |
|---|---|---|
| **Ag-Cu(P)** | 918.3 | 921.0 |
| **$N_2SN$** | 915.8 | 918.4 |
| **$N_3N$** | 916.0 | 918.7 |
| **$C_2N$** | 915.9 | 918.2 |
| **$C_3$** | 915.9 | 918.2 |
| **$H_2O_2$-Ag-Cu** | 917.5 | —— |

**Supplementary Table 5. Summary of the EXAFS data.** $E_0$ and corresponding oxidation states (δ) of Cu.

| Samples | Cu foil | P | $C_3$ | $C_2N$ | $N_3N$ | $N_2SN$ | $Cu_2O$ |
|---|---|---|---|---|---|---|---|
| **$E_0$** | 8979 | 8979.08 | 8979.1 | 8979.2 | 8979.7 | 8979.8 | 8980.5 |
| **δ** | 0 | +0.05 | +0.07 | +0.13 | +0.47 | +0.53 | +1 |

**Supplementary Table 6. EXAFS fitting parameters at the Cu K-edge for different samples ($S_0^2$=0.831).**

| Sample | Shell | $N^a$ | $R$(Å)$^b$ | $\sigma^2$(Å$^2$)$^c$ | $\Delta E_0$ (eV)$^d$ | *R* factor |
|---|---|---|---|---|---|---|
| Cu foil | Cu-Cu | 12.0 | 2.54 | 0.0088 | 5.1 | 0.0005 |
| | Cu-O | 2.0 | 1.85 | 0.0038 | 9.0 | |
| $Cu_2O$ | Cu-Cu | 12.0 | 3.04 | 0.0213 | 11.9 | 0.0033 |
| | Cu-O | 3.8 | 3.58 | 0.0129 | 11.9 | |
| $N_2SN$ | Cu-N | 1.1 | 1.85 | 0.0094 | 7.6 | 0.0041 |
| | Cu-Cu | 5.6 | 2.54 | 0.0085 | 5.9 | |
| $C_2N$ | Cu-Cu | 10.8 | 2.54 | 0.0089 | 5.2 | 0.0005 |
| $N_3N$ | Cu-N | 0.7 | 1.86 | 0.0036 | 9.8 | 0.0015 |
| | Cu-Cu | 6.7 | 2.54 | 0.0051 | 5.6 | |
| $C_3$ | Cu-Cu | 10.4 | 2.54 | 0.0088 | 4.3 | 0.0004 |
| Pristine | Cu-Cu | 11.1 | 2.54 | 0.0097 | 5.0 | 0.0005 |

$^a N$: coordination numbers; $^b R$: bond distance; $^c \sigma^2$: Debye-Waller factors; $^d$ $\Delta E_0$: the inner potential correction. *R* factor: goodness of fit. $S_0^2$ was set to 0.831, according to the experimental EXAFS fit of Cu foil reference by fixing coordination numbers as the known crystallographic value.

**Supplementary Table 7. Estimated ratios (in peak area) between the atop CO and the bridge CO obtained from the deconvoluted spectra[1,2].** The data represents the average values obtained from two independent sets of samples.

| Electrode | Ratio (atop/bridge) |
|---|---|
| $N_2SN$ | 0.51 |
| $N_3N$ | 0.32 |
| $C_2N$ | 2.78 |
| $C_3$ | 0 |
| P | 0.87 |

**Supplementary Table 8. Summary of the estimated FEs for different groups functionalized Ag-Cu electrodes measured at different applied potentials in the H-cell reactors.** The standard deviation of the measurements was estimated from three independent samples.

| Catalysts | Potential vs. RHE (V) | $FE_{C2+}$(%) | $FE_{C1}$(%) | $FE_{H2}$(%) |
|---|---|---|---|---|
| | -1.4 | 37.6±1.8 | 18.3±2.3 | 36.9±2.2 |
| | -1.3 | 47.0±0.9 | 23.3±3.1 | 30.7±2.1 |
| | -1.2 | 57.3±1.4 | 24.9±1.2 | 22.8±2.2 |
| | -1.1 | 47.8±0.6 | 27.6±0.8 | 20.9±2.5 |
| | -1.0 | 43.6±0.8 | 30.5±0.6 | 18.7±1.7 |
| | -0.9 | 39.1±0.6 | 36.3±0.7 | 17.1±0.7 |
| | -0.8 | 38.4±1.9 | 40.1±0.5 | 16.2±0.5 |
| $N_2SN$ | -0.7 | 32.5±1.9 | 38.2±0.6 | 19.0±1.4 |
| | -0.6 | 25.5±2.7 | 32.6±2.3 | 29.5±0.9 |
| | -0.5 | 16.6±1.2 | 24.6±0.6 | 45.2±0.5 |
| | -0.4 | 8.9±1.1 | 19.1±1.5 | 45.6±1.1 |
| | -0.3 | 3.8±0.8 | 13.9±1.2 | 58.8±1.4 |
| | -1.4 | 35.1±1.4 | 9.8±0.5 | 34.9±1.8 |
| | -1.3 | 43.2±2.5 | 12.9±1.2 | 28.9±1.8 |

| | | | | |
|---|---|---|---|---|
| | -1.2 | 52.8±1.5 | 16.6±0.2 | 25.0±0.9 |
| | -1.1 | 49.1±1.6 | 24.6±0.9 | 23.8±0.6 |
| | -1.0 | 44.9±1.8 | 31.4±0.7 | 20.7±1.2 |
| | -0.9 | 39.2±1.0 | 36.6±0.9 | 17.7±0.7 |
| **$N_3N$** | -0.8 | 34.7±0.7 | 43.3±0.8 | 16.3±1.2 |
| | -0.7 | 28.5±0.5 | 43.7±2.2 | 23.8±4.0 |
| | -0.6 | 19.4±1.0 | 35.3±1.7 | 32.4±2.8 |
| | -0.5 | 12.2±0.9 | 28.1±0.6 | 42.9±3.1 |
| | -0.4 | 6.3±0.3 | 24.2±1.2 | 52.1±1.7 |
| | -0.3 | 3.5±1.2 | 28.5±1.2 | 61.9±0.8 |
| | -1.4 | 5.6±1.5 | 5.3±0.3 | 86.5±1.2 |
| | -1.3 | 6.1±0.9 | 6.2±0.2 | 84.3±1.0 |
| | -1.2 | 5.8±0.5 | 6.8±0.3 | 83.5±0.6 |
| | -1.1 | 4.2±0.6 | 8.2±0.1 | 81.2±0.6 |
| | -1.0 | 3.1±0.4 | 9.2±0.1 | 78±0.7 |
| **$C_2N$** | -0.9 | 0.6±0.7 | 12.9±0.2 | 73.9±0.8 |
| | -0.8 | 0 | 16.3±1.0 | 71.3±0.8 |
| | -0.7 | 0 | 16.7±1.5 | 75.0±0.6 |
| | -0.6 | 0 | 13.2±1.0 | 77.9±2.0 |
| | -0.5 | 0 | 10.7±0.6 | 80.8±1.1 |
| | -0.4 | 0 | 5.3±1.2 | 84.4±0.8 |
| | -0.3 | 0 | 0 | 90.6±0.8 |
| | -1.4 | 2.1±1.1 | 7.1±0.3 | 86.0±2.5 |
| | -1.3 | 4.4±0.2 | 9.6±1.4 | 83.8±0.9 |
| | -1.2 | 5.0±0.4 | 10.8±1.2 | 81.8±1.2 |
| | -1.1 | 2.4±0.3 | 13.5±1.2 | 80.1±0.7 |
| | -1.0 | 1.6±0.3 | 16.6±2.9 | 74.9±2.1 |
| | -0.9 | 0 | 18.3±1.5 | 75.0±0.5 |
| **$C_3$** | -0.8 | 0 | 16.6±1.7 | 75.3±1.1 |
| | -0.7 | 0 | 9.9±2.5 | 79.3±5.2 |
| | -0.6 | 0 | 6.2±1.9 | 87.6±2.1 |
| | -0.5 | 0 | 3.3±1.5 | 88.2±3.2 |
| | -0.4 | 0 | 1.2±0.4 | 86.3±6.2 |
| | -0.3 | 0 | 0.4±0.5 | 91.7±3.6 |
| | -1.4 | 16.6±0.4 | 24.6±1.3 | 55.2±1.9 |
| | -1.3 | 18.5±1.2 | 32.2±0.7 | 45.7±2.8 |
| | -1.2 | 18.3±0.5 | 33.9±0.5 | 38.2±2.2 |

| | | | | |
|---|---|---|---|---|
| | -1.1 | 14.7±2.0 | 31.6±0.7 | 35.7±1.2 |
| | -1.0 | 12.4±1.3 | 29.9±0.6 | 34.2±0.6 |
| | -0.9 | 8.2±0.7 | 32.9±0.7 | 32.6±0.7 |
| | -0.8 | 4.7±0.7 | 26.4±1.1 | 42.8±0.5 |
| **P** | -0.7 | 2.5±0.8 | 19.3±2.6 | 52.8±1.3 |
| | -0.6 | 1.8±1.1 | 12.7±1.2 | 63.5±0.9 |
| | -0.5 | 0 | 8.9±0.5 | 73.9±0.9 |
| | -0.4 | 0 | 4.5±0.4 | 85.9±0.7 |
| | -0.3 | 0 | 1.7±0.4 | 93.4±1.4 |

**Supplementary Table 9. Summary of the FEs obtained on the different functionalized Ag-Cu electrodes measured at different applied potentials in the MEA electrolyzers.** The standard deviation of the measurements was estimated from three independent samples.

| **Catalysts** | **Voltage (V)** | **$FE_{C2+}$(%)** | **$FE_{C1}$(%)** | **$FE_{H2}$(%)** |
|---|---|---|---|---|
| | -2.8 | 24.6±4.1 | 15.8±4.7 | 44.8±3.5 |
| | -2.9 | 30.7±3.1 | 14.8±4.9 | 41.4±3.6 |
| | -3.0 | 34.3±2.7 | 15.2±5.5 | 36.7±3.0 |
| | -3.1 | 34.9±3.1 | 15.1±5.6 | 28.9±1.0 |
| | -3.2 | 36.4±4.5 | 15.1±5.5 | 27.8±2.6 |
| | -3.3 | 42.5±1.8 | 16.2±6.9 | 25.9±3.6 |
| | -3.4 | 44.9±3.8 | 14.5±4.2 | 25.2±2.5 |
| | -3.5 | 47.6±3.1 | 15.6±3.9 | 23.2±3.4 |
| | -3.6 | 50.8±2.2 | 17.1±3.6 | 23.3±3.2 |
| | -3.7 | 53.0±3.3 | 18.0±2.5 | 22.7±3.2 |
| | -3.8 | 54.8±2.9 | 18.4±1.2 | 23.1±3.3 |
| | -3.9 | 56.4±2.2 | 19.3±1.5 | 20.4±3.1 |
| | -4.0 | 58.7±2.0 | 18.1±2.5 | 20.6±1.8 |
| **$N_2$SN** | -4.1 | 59.9±1.9 | 17.0±2.0 | 19.1±0.5 |
| | -4.15 | 62.5±1.8 | 16.7±2.8 | 19.5±0.1 |
| | -4.2 | 64.4±2.9 | 16.1±3.5 | 18.0±0.6 |
| | -4.25 | 67.5±2.2 | 14.6±2.9 | 16.9±0.7 |
| | -4.3 | 71.2±2.7 | 14.1±3.4 | 16.5±1.2 |

| | | | | |
|---|---|---|---|---|
| | -4.35 | 74.2±3.3 | 13.6±3.8 | 15.8±1.4 |
| | -4.4 | 77.8±4.3 | 12.4±2.9 | 15.7±1.4 |
| | -4.45 | 78.2±3.5 | 10.5±2.4 | 15.0±1.4 |
| | -4.5 | 79.9±2.3 | 8.0±0.5 | 13.9±0.5 |
| | -4.55 | 78.5±1.7 | 8.2±1.4 | 15.9±0.6 |
| | -4.6 | 74.9±1.6 | 8.7±2.8 | 18.5±1.8 |
| | -4.65 | 71.0±0.9 | 8.3±3.3 | 20.4±1.1 |
| | -4.7 | 65.9±0.9 | 8.0±3.5 | 25.0±4.4 |
| | -4.75 | 61.0±1.1 | 8.4±4.2 | 27.7±3.4 |
| | -4.8 | 57.2±3.4 | 8.1±4.3 | 31.6±4.5 |

| **Catalysts** | **Voltage (V)** | **$FE_{C2+}$(%)** | **$FE_{C1}$(%)** | **$FE_{H2}$(%)** |
|---|---|---|---|---|
| | -2.8 | 1.6±1.0 | 26.6±3.1 | 56.3±3.4 |
| | -2.9 | 6.4±4.3 | 28.4±3.2 | 52.4±1.1 |
| | -3.0 | 14.4±3.9 | 30.5±3.8 | 45.9±1.7 |
| | -3.1 | 21.1±3.5 | 31.4±2.7 | 41.9±2.1 |
| | -3.2 | 25.1±3.9 | 28.6±1.0 | 39.8±3.0 |
| | -3.3 | 29.4±5.3 | 26.8±0.5 | 35.7±4.6 |
| | -3.4 | 32.5±0.8 | 26.1±0.9 | 33.1±4.2 |
| | -3.5 | 41.6±1.9 | 23.5±0.6 | 30.7±5.1 |
| | -3.6 | 46.9±2.7 | 21.4±1.5 | 30.1±1.8 |
| | -3.7 | 52.1±1.7 | 19.9±2.6 | 28.3±1.5 |
| | -3.8 | 54.4±2.0 | 19.4±2.6 | 26.7±1.9 |
| | -3.9 | 55.6±3.9 | 16.6±3.0 | 21.2±1.3 |
| | -4.0 | 58.1±5.3 | 16.5±2.6 | 20.9±0.9 |
| **$N_3N$** | -4.1 | 57.7±3.6 | 17.8±3.9 | 20.7±1.1 |
| | -4.15 | 58.1±3.1 | 17.3±3.5 | 20.7±1.4 |
| | -4.2 | 59.8±3.9 | 17.3±3.2 | 19.6±0.8 |
| | -4.25 | 61.7±4.0 | 18.2±2.8 | 18.8±0.6 |
| | -4.3 | 62.8±5.0 | 17.6±2.3 | 18.0±0.8 |
| | -4.35 | 64.1±4.9 | 17.2±1.9 | 17.2±0.7 |
| | -4.4 | 65.9±4.3 | 16.4±1.5 | 16.5±0.6 |
| | -4.45 | 66.9±3.7 | 15.3±0.8 | 16.7±0.2 |
| | -4.5 | 68.4±3.9 | 14.6±0.7 | 17.9±0.9 |

| | | | | |
|---|---|---|---|---|
| | -4.55 | 68.9±5.9 | 12.9±1.0 | 20.0±1.6 |
| | -4.6 | 67.6±4.9 | 11.2±2.2 | 23.3±1.4 |
| | -4.65 | 63.5±3.8 | 10.0±2.0 | 25.7±0.7 |
| | -4.7 | 60.6±2.1 | 9.1±2.3 | 28.7±0.3 |
| | -4.75 | 56.4±1.5 | 8.5±2.5 | 32.3±0.6 |
| | -4.8 | 52.2±1.4 | 8.1±2.2 | 35.9±1.2 |

| **Catalysts** | **Voltage (V)** | **$FE_{C2+}$(%)** | **$FE_{C1}$(%)** | **$FE_{H2}$(%)** |
|---|---|---|---|---|
| | -2.8 | 0 | 13.2±1.2 | 77.3±0.6 |
| | -2.9 | 0 | 17.1±1.8 | 75.7±0.6 |
| | -3.0 | 0 | 20.1±1.0 | 74.2±1.3 |
| | -3.1 | 3.2±0.5 | 24.3±1.4 | 72.0±2.3 |
| | -3.2 | 5.5±0.8 | 26.1±1.4 | 70.2±2.9 |
| | -3.3 | 6.9±1.7 | 29.5±0.8 | 68.0±2.2 |
| | -3.4 | 6.7±1.7 | 31.5±0.8 | 66.7±3.5 |
| | -3.5 | 7.4±1.8 | 29.5±0.6 | 63.4±3.8 |
| | -3.6 | 7.2±1.7 | 29.1±0.7 | 61.1±4.0 |
| | -3.7 | 7.9±2.1 | 27.5±0.7 | 58.6±3.4 |
| | -3.8 | 9.2±2.2 | 25.3±1.5 | 55.7±3.3 |
| | -3.9 | 10.8±2.8 | 23.7±1.8 | 55.2±2.7 |
| | -4.0 | 12.3±2.4 | 22.0±1.4 | 52.8±2.5 |
| **$C_2N$** | -4.1 | 12.7±2.3 | 19.7±0.9 | 50.3±2.5 |
| | -4.15 | 14.6±2.3 | 17.5±0.4 | 48.1±2.5 |
| | -4.2 | 16.9±2.7 | 14.5±0.7 | 46.0±1.9 |
| | -4.25 | 18.2±3.4 | 12.7±0.8 | 44.3±1.8 |
| | -4.3 | 20.7±2.6 | 11.4±1.3 | 41.8±1.6 |
| | -4.35 | 21.9±1.8 | 9.5±1.9 | 39.9±1.1 |
| | -4.4 | 23.1±1.7 | 7.7±1.4 | 39.4±0.8 |
| | -4.45 | 24.6±1.3 | 6.3±1.3 | 41.8±0.2 |
| | -4.5 | 26.1±0.7 | 5.8±1.3 | 44.3±1.0 |
| | -4.55 | 29.3±0.7 | 4.8±0.6 | 47.7±1.6 |
| | -4.6 | 30.3±1.5 | 4.2±0.3 | 50.1±1.7 |
| | -4.65 | 28.6±0.9 | 3.9±0.6 | 53.6±1.8 |
| | -4.7 | 27.7±0.8 | 3.3±0.3 | 56.7±2.5 |
| | -4.75 | 26.4±0.4 | 3.3±0.3 | 58.5±2.7 |
| | -4.8 | 24.7±0.1 | 2.7±0.2 | 62.1±1.7 |

| **Catalysts** | **Voltage (V)** | **$FE_{C2+}$(%)** | **$FE_{C1}$(%)** | **$FE_{H2}$(%)** |
|---|---|---|---|---|
| | -2.8 | 0 | 7.1±2.0 | 82.1±1.3 |
| | -2.9 | 0 | 10.9±2.1 | 79.2±1.7 |
| | -3.0 | 0 | 13.7±2.2 | 77.6±1.4 |
| | -3.1 | 0 | 15.9±0.9 | 76.3±1.8 |
| | -3.2 | 0 | 17.4±0.4 | 74.1±1.8 |
| | -3.3 | 0 | 19.2±0.1 | 72.2±1.8 |
| | -3.4 | 0 | 18.0±0.1 | 70.9±3.4 |
| | -3.5 | 1.5±0.4 | 17.3±0.3 | 68.7±3.6 |
| | -3.6 | 2.2±0.2 | 17.3±1.1 | 67.9±3.3 |
| | -3.7 | 2.9±0.1 | 16.2±1.3 | 65.9±2.8 |
| | -3.8 | 4.4±0.2 | 15.3±1.5 | 64.4±3.3 |
| | -3.9 | 4.7±0.4 | 14.2±1.3 | 62.5±3.2 |
| | -4.0 | 6.9±0.2 | 14.1±1.8 | 60.8±2.8 |
| **$C_3$** | -4.1 | 7.7±0.6 | 13.5±1.9 | 57.5±2.7 |
| | -4.15 | 9.8±0.6 | 12.5±1.5 | 56.9±2.5 |
| | -4.2 | 10.1±0.3 | 11.5±1.7 | 55.6±2.5 |
| | -4.25 | 10.9±0.9 | 10.5±2.0 | 53.2±2.4 |
| | -4.3 | 11.9±0.6 | 9.4±2.1 | 50.5±1.5 |
| | -4.35 | 13.9±0.3 | 8.6±2.1 | 48.7±1.1 |
| | -4.4 | 15.6±0.8 | 7.7±2.0 | 46.9±0.8 |
| | -4.45 | 16.8±0.6 | 6.7±1.9 | 48.3±0.7 |
| | -4.5 | 17.5±0.3 | 5.9±1.8 | 49.6±0.9 |
| | -4.55 | 19.6±0.2 | 4.9±1.4 | 51.4±1.0 |
| | -4.6 | 21.4±0.3 | 4.1±0.8 | 53.3±0.4 |
| | -4.65 | 20.2±0.2 | 3.4±0.6 | 55.5±0.4 |
| | -4.7 | 19.0±0.6 | 2.9±0.2 | 57.7±0.9 |
| | -4.75 | 17.3±0.6 | 2.4±0.5 | 60.3±1.1 |
| | -4.8 | 15.6±0.5 | 2.3±0.1 | 65.2±1.0 |

| **Catalysts** | **Voltage (V)** | **$FE_{C2+}$(%)** | **$FE_{C1}$(%)** | **$FE_{H2}$(%)** |
|---|---|---|---|---|
| | -2.8 | 0 | 15.5±0.6 | 70.8±1.9 |
| | -2.9 | 2.7±1.9 | 19.9±2.0 | 67.6±0.7 |
| | -3.0 | 5.7±0.1 | 24.2±2.3 | 64.8±1.3 |
| | -3.1 | 7.2±0.8 | 27.1±1.4 | 61,5±0.8 |
| | -3.2 | 9.7±1.0 | 31.9±2.4 | 56.5±1.9 |

| | | | | |
|---|---|---|---|---|
| | -3.3 | 11.8±0.8 | 33.8±1.4 | 52.3±2.2 |
| | -3.4 | 13.4±1.0 | 36.6±1.9 | 49.4±2.4 |
| | -3.5 | 15.5±1.3 | 39.0±1.2 | 46.9±1.2 |
| | -3.6 | 15.9±1.1 | 37.6±1.2 | 44.2±1.6 |
| | -3.7 | 15.6±1.7 | 33.3±2.2 | 40.2±1.0 |
| | -3.8 | 16.1±2.1 | 31.9±0.7 | 38.6±1.4 |
| | -3.9 | 16.7±1.0 | 30.0±0.9 | 37.6±0.9 |
| | -4.0 | 18.0±1.7 | 28.1±1.1 | 38.2±1.2 |
| **Pristine (P)** | -4.1 | 17.6±1.7 | 24.4±1.9 | 38.4±1.2 |
| | -4.15 | 18.9±1.7 | 24.6±0.9 | 38.6±0.5 |
| | -4.2 | 21.3±2.3 | 23.7±1.1 | 40.5±1.0 |
| | -4.25 | 21.5±1.4 | 23.3±1.1 | 41.8±0.8 |
| | -4.3 | 22.3±1.0 | 22.0±1.3 | 42.3±0.7 |
| | -4.35 | 23.8±1.0 | 21.1±1.2 | 43.8±0.8 |
| | -4.4 | 26.8±1.1 | 20.7±1.1 | 45.1±1.1 |
| | -4.45 | 28.7±1.1 | 21.9±1.2 | 45.2±1.4 |
| | -4.5 | 30.9±2.1 | 22.6±1.7 | 46.3±1.5 |
| | -4.55 | 32.4±0.8 | 22.5±1.8 | 46.9±1.6 |
| | -4.6 | 33.9±0.9 | 24.0±2.3 | 48.0±1.9 |
| | -4.65 | 32.9±1.2 | 23.1±2.7 | 48.8±1.5 |
| | -4.7 | 32.5±0.8 | 22.2±2.8 | 49.4±1.4 |
| | -4.75 | 29.9±0.4 | 18.8±2.5 | 55.4±1.5 |
| | -4.8 | 27.8±1.1 | 16.0±1.6 | 64.3±1.3 |

## References


1 Fielicke, A., Gruene, P., Meijer, G. & Rayner, D. M. The adsorption of CO on transition metal clusters: A case study of cluster surface chemistry. *Surf Sci* **603**, 1427-1433 (2009).

2 Li, F. *et al.* Molecular tuning of $CO_2$-to-ethylene conversion. *Nature* **577**, 509-513 (2020).
